# What is the effect of active ingredients in dentifrice on inhibiting the regrowth of overnight plaque? A systematic review

**DOI:** 10.1111/idh.12423

**Published:** 2019-12-22

**Authors:** Cees Valkenburg, Dagmar Else Slot, GA (Fridus) Van der Weijden

**Affiliations:** ^1^ General Dentist and Clinical Epidemiologist Hoevelaken The Netherlands; ^2^ Department of Periodontology Academic Centre for Dentistry Amsterdam (ACTA) University of Amsterdam and Vrije Universiteit Amsterdam Amsterdam The Netherlands

**Keywords:** dentifrice, oral hygiene, plaque, plaque inhibition, plaque regrowth, systematic review, toothbrushing, toothpaste

## Abstract

**Objectives:**

The aim of this systematic review was to establish the adjuvant clinical effect of brushing with a dentifrice containing purported active ingredients as compared to a regular sodium fluoride dentifrice with respect to the inhibition of overnight dental plaque regrowth from studies with human participants.

**Methods:**

MEDLINE‐PubMed, EMBASE and Cochrane CENTRAL were searched, up to June 2019. The inclusion criteria were controlled clinical trials with participants aged ≥ 18 years in good general health. Studies were included that evaluated the effect of toothbrushing with a dentifrice on the inhibition of overnight dental plaque regrowth when an active ingredient was added to the dentifrice as compared to a common sodium fluoride product. Data were extracted from the eligible studies, the risk of bias was assessed, and a meta‐analysis was performed where feasible.

**Result:**

Independent screening of 213 unique papers resulted in 10 eligible publications that provided 14 comparisons. Stannous fluoride and triclosan dentifrices were found as the active ingredients. The descriptive analysis indicated that all, but two comparisons demonstrated an additional effect on the active‐ingredient dentifrice. The meta‐analysis supported and strengthened these findings. It showed that when plaque was scored digitally, a DiffM was −3.15(95% CI [−4.61:‐1.69], *P* < .001, prediction interval [−5.07;‐1.24]). When plaque was scored clinically, the difference of means (DiffM) was −0.33(95% CI [−0.49:‐0.16], *P* < .001, prediction interval [−0.87; 0.21]).

**Conclusion:**

The results of this review demonstrate moderate‐quality evidence that brushing with an active‐ingredient dentifrice with stannous fluoride or triclosan does provide an added clinically relevant effect concerning plaque inhibition capabilities that surpass the effect of a regular sodium fluoride dentifrice.

## 1 INTRODUCTION

Routine toothbrushing is perhaps the single most important step for an individual to take in order to reduce plaque accumulation and to reduce the consequent risk of plaque‐associated diseases, such as periodontitis and caries.^1^ In addition to toothbrushing, dentifrices can help with stain removal, breath freshening and provide a feeling of cleanliness.^2^ Fluoride‐containing dentifrices also play an essential role in caries prevention.^3^, ^4^ A recent systematic review concluded that dentifrice does not provide an added effect for the mechanical removal of dental plaque. In terms of plaque removal, toothbrushing is at least as effective as toothbrushing with a dentifrice.^5^ In this respect, it seemed that the mechanical action provided by the toothbrush was the main factor in the plaque removal process.^6^ However, despite brushing every day, people are typically not effective brushers and live with large amounts of plaque on their teeth.^7^ It is here that chemical adjuncts to toothbrushing could be beneficial. Chemicals could prevent bacterial attachment, stop bacterial division and plaque growth, or may even remove plaque.^8^

Although the long‐term use of dentifrices with active ingredients intended for patients with gingivitis is associated with the prevention of bacterial biofilm formation, only a few of these products have been systematically evaluated in relation to gingival health. For example, the use of stannous fluoride or triclosan dentifrices resulted in greater gingivitis and plaque reduction than the use of a conventional dentifrice.^9^, ^10^, ^11^, ^12^, ^13^, ^14^ However, the primary aim of these studies had a focus on plaque removal and not on preventing plaque accumulation on the dentition. An important intervention target for chemotherapeutics is to optimize plaque control by inhibiting overnight plaque regrowth.^15^

Since the use of dentifrices is widespread and available scientific literature suggests that dentifrices reduce plaque regrowth,^16^ a further aspect of interest is whether following a brushing exercise dentifrices that contain purported active ingredients reduce overnight plaque regrowth more than regular sodium fluoride dentifrices. This overnight model was not included in a recent SR which demonstrated moderate‐quality evidence in a 4‐day non‐brushing model with dentifrice slurry for a weak inhibitory effect on plaque regrowth in favour of the use of a dentifrice intended for daily use.^16^

Therefore, the purpose of this paper was to systematically and critically appraise the literature concerning the adjuvant effect of a dentifrice on the inhibition of overnight plaque regrowth.

## 2 MATERIALS AND METHODS

This systematic review was prepared and described in accordance with the Cochrane Handbook for Systematic Reviews of Interventions^17^ and in the guidelines Transparent Reporting of Systematic Reviews and Meta‐Analyses (PRISMA statement).^18^

### 2.1 Protocol development

The protocol for this review was developed “a priori” and registered with the International Prospective Register of Systematic Reviews^19^ under the registration number CRD42019126734. All post hoc changes were appropriately noted (see Appendix S14).

### 2.2 Focused PICOS question

In healthy adults (P), what is the effect of brushing with a dentifrice containing purported active ingredients to inhibit overnight plaque regrowth (I) compared to a regular sodium fluoride dentifrice (C) according the clinical indices of dental plaque (O) using an overnight plaque accumulation model (S)?

### 2.3 Search strategy

A structured search strategy was designed to retrieve all relevant studies. As proposed in the Cochrane Handbook the National Library of Medicine, Washington, DC (MEDLINE‐PubMed), EMBASE and the Cochrane Central Register of Controlled Trials (CENTRAL) were searched from initiation to June 2019 for appropriate papers that answered the focused question. The reference lists of the studies included were hand‐searched to identify additional potentially relevant studies, and Google Scholar was used as additional source. No limitations were placed on language or date of publication in the electronic searches of the databases. For details regarding the search terms used, see Table 1.

**Table 1:** Search terms used for PubMed‐MEDLINE and Cochrane CENTRAL. The search strategy was customized according to the database being searched. The following strategy was used in the search: ( [<intervention>] AND [<outcome>])

([ <intervention: toothpaste>
([*MeSH terms/all subheadings*] toothpastes)
OR
([*text words*] toothpaste OR dentifrice OR toothpastes OR dentifrices)]
AND
[ <outcome: overnight dental plaque>
(([text words] overnight) AND
([*MeSH terms/all subheadings*] dental plaque OR dental plaque index OR dental deposits))
OR
([*text words*] plaque OR plaque removal OR plaque index OR dental plaque OR dental)])

### 2.4 Screening and selection

The titles and abstracts of the studies obtained from the searches were screened independently by two reviewers (CV and DES) to select studies that potentially met the inclusion criteria. No language restrictions were imposed. Based on the title and abstract, the full‐text versions of potentially relevant papers were obtained. These papers were categorized (CV and DES) as definitely eligible, definitely not eligible or questionable. Disagreements concerning eligibility were resolved by referring to the original article. If no consensus could be reached, the decision was resolved through arbitration by a third reviewer (GAW). The papers that fulfilled all inclusion criteria were processed for data extraction.

### 2.5 The inclusive and exclusive criteria

#### 2.5.1 Inclusion criteria

The included studies were considered to meet the following criteria:

(a) The study design was either a randomized controlled clinical trial (RCT) or a controlled clinical trial (CCT), and.

(b) the publications were available as full reports.

Population:

(c) The studies were conducted with humans, who were not institutionalized and who were 18 years of age or older,

(d) in good general health (no systematic disorders), and without orthodontic appliances and/or removable prostheses.

Intervention:

(e) The intervention was toothbrushing with an active‐ingredient dentifrice.

Comparison:

(f) The control was a standard fluoride dentifrice.

Outcome:

(g) The studies evaluated regrowth of plaque.

(h) Setting: Overnight plaque accumulation model.

#### 2.5.2 Exclusion criteria

*Chlorhexidine was the active ingredient incorporated in a dentifrice.^20^

*Additionally, rinsing with an antiseptic as part of the intervention or control regimen.

For details, see Appendix S2.

### 2.6 Assessment of heterogeneity

The following factors were used to evaluate the heterogeneity of the outcomes of the different studies: study design, participant characteristics, study group details, side effects and industry funding.

### 2.7 Assessment of methodological quality and risk of bias

All included studies were independently scored for their methodological quality by two reviewers (CV and DES) using the checklist presented in Appendix S3. Disagreement was resolved by consensus, and if disagreement persisted, the decision was resolved through arbitration by a third reviewer (GAW). The assessed items as detailed in Appendix S3 were used to classify a study as having an estimated low, moderate or high risk of bias.^21^

### 2.8 Data extraction

The characteristics of the population, intervention, comparison and outcomes were extracted independently from all studies by two reviewers (CV and DES) using a specially designed data extraction form. Means and standard deviations (SDs) were extracted. Some studies provided standard errors (SEs) of the means. Where possible, the authors calculated standard deviation based on the sample size (SE = SD/√N) and transformed logarithmic value back to the raw scale.^22^ For those papers that provided insufficient data to be included in the analysis, the first or corresponding author was contacted to request additional data. If no response was received within a reasonable amount of time, the study was not included in the meta‐analysis.

### 2.9 Data analysis

As a summary, a descriptive data presentation was used for all studies. For studies that had multiple treatment arms and for which data from the control group were compared with more than one other group, the number of participants (n) in the control group was divided by the number of comparisons. The data are presented, and the modifications of the original indices^23^, ^24^, ^25^ are provided. The difference of means (DiffM) between brushing with and without an active‐ingredient dentifrice was calculated using a “random‐effects” model with an “inverse variance” method as proposed by DerSimonian and Laird.^26^ For MA with more than two comparisons, 95% predictive intervals were calculated to quantify potential treatment effects in a future clinical setting.^27^

Heterogeneity was tested using the chi‐square test and the *I* ^2^ statistic with 95% confidence intervals around *I* ^2^.^17^, ^28^ If possible, tests for small‐study effects were conducted in order to detect potential publication bias. Therefore, regression tests and their modifications as proposed by Egger et al^29^ and Sterne et al^30^ were used as well as non‐parametric tests and their modifications, as proposed by Begg et al,^31^ and the trim‐and‐fill method and their modifications, as proposed by Duval and Tweedie^32^ and Peters et al^33^. The adjusted treatment effect estimate as based on the Copa selection model was calculated.^34^, ^35^ Trial sequential analysis (TSA) was carried out which allow for an estimate of the risk of a type Ι error. The required information size (RIS) and the trial sequential monitoring boundaries (TSMB) for benefit or futility were calculated. The RIS was calculated based on a type Ι error risk of α = 5% and a type ΙΙ error risk of β = 0.20, with a statistical test power of 80%. RIS was accounted for heterogeneity and multiple comparisons. The Lan‐DeMets version^36^ of the O’Brien‐Fleming function^37^ was used for calculating the TSMBs. TSA software version 0.9.5.10 Beta (Copenhagen Trial Unit, Copenhagen, Denmark) was used.^38^, ^39^, ^40^, ^41^

Inflation bias or “p‐hacking” was tested with a P‐curve analysis.^42^, ^43^ Post hoc sensitivity analysis was conducted to evaluate the influence of a single study on the overall effect estimate by stepwise omitting, one by one, each of the studies included in the meta‐analysis and re‐evaluating the summary effect estimates.^44^, ^45^, ^46^ Post hoc analysis was conducted on study design. Computations for the MA were performed using R (https://www.r-project.org) with the packages meta^44^, ^47^ and metafor.^48^

In order to judge the clinical relevance of study results, “distribution‐based” methods were used.^49^, ^50^, ^51^, ^52^, ^53^ The clinical relevance was scored as not clinically relevant, potentially clinically relevant or clinically relevant^51^, ^52^ based on the relationship among the mean difference of the variable, minimal important differences (MIDs) and effect sizes (ES). The MID was determined by multiplying the effect size of the difference obtained between groups considered as important (0.2 or 0.5 ES according to Cohen) by the pooled baseline standard deviation between the two groups.^51^, ^54^

### 2.10 Grading the “body of evidence”

The Grading of Recommendations Assessment, Development and Evaluation (GRADE) system was used to rank the evidence.^55^ Two reviewers (DES and GAW) rated the quality of the evidence and the strength and direction of the recommendations^56^ according to the following aspects: risk of bias, consistency of results, directness of evidence, precision and publication bias, and magnitude of the effect.

## 3 RESULTS

### 3.1 Search and selection results

**Figure 1:**
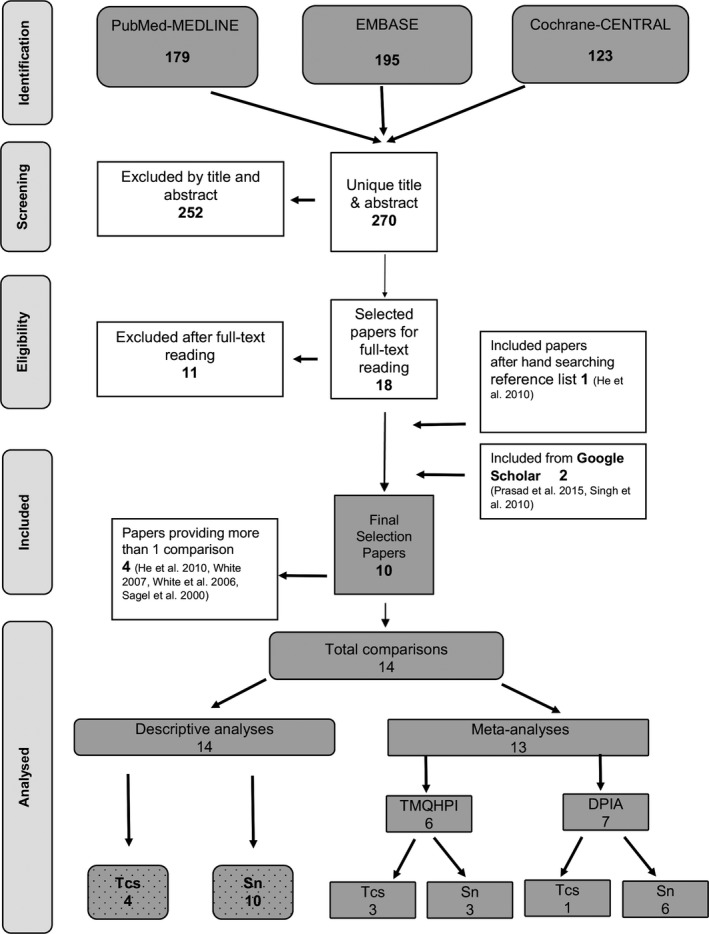
Search flow chart

The search of the MEDLINE‐PubMed and Cochrane CENTRAL databases resulted in 270 unique papers (for details, see Appendix S1). Screening of the titles and abstracts resulted in 18 papers, for which full reports were obtained. After detailed reading of the full reports, 11 studies were excluded at this stage. Manual searching of the reference lists of the selected papers provided one additional relevant paper. Additional searching in Google Scholar revealed two additional suitable papers. In total, 10 eligible publications describing 14 comparisons were included in this systematic review. For details, see Figure 1.

### 3.2 Study characteristics and heterogeneity

The included studies exhibited considerable heterogeneity. Information regarding the study characteristics is presented in detail in Appendix S2A. The demographic characteristics are summarized in Appendix S2B.

With the exception of two studies,^25^, ^57^ all reported to be randomized. Only two studies had a parallel design.^58^, ^59^ The others all used a crossover design. All the studies included evaluated overnight plaque scores in the morning before brushing. In one study, the period of no oral hygiene extended to 12 hours^59^ and, in two other studies, to 24 hours.^57^, ^60^ A fourth study used a power brush.^61^

The studies were carried out in several countries around the world, which included India,^59^ China,^62^ the UK^60^, ^61^ and the United States.^25^, ^57^, ^58^, ^63^, ^64^

A total of 456 participants provided data for this review. Participants ranged from experienced employee dentifrice panels^57^, ^61^, ^63^, ^64^, ^65^ to non‐dental populations.^58^, ^59^, ^62^

In the studies, there were several dropouts. Two participants did not complete the study protocol,^62^, ^64^ and 15 participants were ineligible due to migration, unforeseen health events and other unspecified reasons.^59^ One study did not describe dropouts.^58^

The time for brushing varied in the studies. One study instructed participants to brushing for one minute twice per day^58^, ^59^ and two studies instructed participants to brush for two minutes twice per day.^61^ The studies did not allow any additional oral hygiene products but in four studies,^15^, ^61^, ^63^, ^64^ floss users could continue to floss their posterior teeth only. In three studies, participants were explicitly instructed to brush only their lingual surfaces in the evening prior to the next appointment^25^, ^63^, ^66^ and in one study, the participants were requested to swish the intra‐oral dentifrice slurry over the facial surfaces for 30 seconds.^25^ Compliance in using the dentifrice was not monitored in any of the studies. One study^62^ provided, during the initial study visit, a full dental prophylaxis to remove all supragingival plaque and calculus.

The dentifrices used in the studies varied between the studies with regard to the following: percentage fluoride compound, percentage stannous fluoride and the difference in accurately describing the ingredients. One study used a stannous fluoride prototype dentifrice.^63^ All the other dentifrice products were marketed at the time of the individual studies. Information on dentine abrasivity (RDA) was lacking in all studies.

In the included papers, two different indices for plaque scoring were used. Seven studies^25^, ^57^, ^61^, ^63^, ^64^, ^65^, ^66^ used the digital plaque image analysis (DPIA) index,^25^ which evaluates the percentage of visible tooth area that is covered with plaque. Three studies^58^, ^59^, ^62^ used the Quigley‐Hein Index modified by Turesky et al,^24^ which scores plaque on a 0 to 5 point scale. The Turesky et al^24^ index was scored full mouth and the DPIA index scored the facial aspect of the 12 front teeth.^25^

### 3.3 Industry funding

All studies mentioned the utilization of commercially available oral hygiene products (dentifrice, toothbrush). Seven studies^57^, ^61^, ^62^, ^63^, ^64^, ^65^, ^67^ mentioned funding by the Procter and Gamble Company, and one study^58^ mentioned funding by the Colgate‐Palmolive Company. Two studies^25^, ^59^ mentioned no funding, but (some) authors were employees of the Procter and Gamble Company.

### 3.4 Side effects

Three included papers did mention the recording of side effects^25^, ^62^, ^64^ but did not observe any adverse events or side effects.

### 3.5 Methodological quality and assessment of bias

To estimate the potential risk of bias, the methodological qualities of the included studies were used, as assessed in the checklist presented in Appendix S3. The procedures for allocation concealment were not described in any of the selected studies. Two studies provided explicit information on sample size calculation and power.^59^, ^61^ Blinding to the product was described in all the selected studies with the exception of one study.^25^ Blinding of the examiner to the product however was unclear. Based on a summary of the proposed criteria, the estimated potential risk of bias was low for six studies,^58^, ^59^, ^61^, ^62^, ^65^, ^67^ moderate for two studies^63^, ^64^ and high for two studies.^25^, ^57^

### 3.6 Study outcomes results

Appendix S4 presents the results of the data extraction. For plaque scores, the Turesky et al^24^ modification of the Quigley and Hein Plaque Index was assessed clinically, and outcomes according to the DPIA index^25^ were scored digitally.

### 3.7 Descriptive analysis

Appendix S5 provides a descriptive summary of the significant difference between an active‐ingredient dentifrice compared to a regular sodium fluoride dentifrice on overnight dental plaque regrowth, as reported by the original authors. Twelve comparisons out of 14 demonstrated a significant difference between interventions in favour of the use of dentifrice with purported active ingredients, while one comparison with a stannous fluoride–containing dentifrice 59 and one comparison with a triclosan dentifrice^64^ demonstrated no significant difference in comparison with a regular dentifrice.

### 3.8 Meta‐analysis

All studies except one^25^ provided information on sample size, outcomes and standard errors/deviations. No additional data were obtained after contacting the authors. A random meta‐analysis could be performed, but the studies were separately analysed based on the index used. A subgroup analysis was performed by dentifrice ingredient. Table 2 presents the outcomes.

**Table 2:** Meta‐analysis of the clinically and digital overnight morning plaque scores (Turesky Modification of the Quigley‐Hein Plaque Index and DPIA Plaque Index) comparing the efficacy of toothbrushing with the use of an active dentifrice or regular dentifrice

Measurement moment	Included studies		Effect size (random)				Prediction interval^a^	Heterogeneity		For details, see Appendices S1‐ S15
				DiffM	95% CI	*P*‐value	95% PI	*I^2^* 95% CI	*P*‐value^b^	
Baseline (Turesky)	Six comparisons He et al^62^ 2x Prasad et al^15^ 2x Singh et al^58^ 2x	ALL	6	0.07	(−0.01; 0.15)	.093	[−0.05; 0.19]	0% (0‐0%)	.98	S6a
		Tcs	3	0.09	(−0.02; 0.21)	.118	[−0.66; 0.84]	0% (0‐0%)	.93	
		Sn	3	0.05	(−0.07; 0.17)	.422	[−0.72; 0.82]	0% (0‐36%)	.85	
END (Turesky)	Six comparisons He et al^62^ 2x Prasad et al^15^ 2x Singh et al^58^ 2x	ALL	6	−0.33	(−0.49; −0.16)	**<.001**	[−0.87; 0.21]	78% (52‐90%)	**<.01**	S6b Funnel plot S8a and S8b
		Tcs	3	−0.43	(−0.72; −0.13)	**.004**	[−4.01; 3.15]	86% (61‐95%)	**<.01**	
		Sn	3	−0.23	(−0.42; −0.04)	**.016**	[−2.30; 1.84]	63% (0%‐89%)	.07	
END (DPIA)	Seven comparisons Bellamy et al^65^ Bellamy et al^66^ Bellamy et al^61^ White et al^15^ White et al^82^ White et al^64^ 2x	ALL	7	−3.15	(−4.61; −1.69)	**<.001**	(−5.07; −1.24)	0% (0‐45%)	.78	S7 Funnel plot S8a and S8b

Abbreviations: ALL, Triclosan and stannous dentifrice combined; CI, Confidence interval; DiffM, difference of means; *I* ^2^, Heterogeneity test, *I* ^2^ statistic; PI, Prediction interval; Sn, Stannous dentifrice; Tcs, Triclosan dentifrice.

a ≥ 3 comparisons.

b The number of decimals to which the annotations have been rounded off is 2.

The analysis of the available data from the modification of the Quigley and Hein (Q&H) Plaque Index by Turesky et al^24^ included three studies, which resulted in six comparisons. End scores did provide a significant difference of means in favour of the use of dentifrice with stannous fluoride or triclosan (DiffM −0.33; *P* < .001; 95% CI: [−0.49; −0.16]). Appendix S6A,B present the forest plot of baseline and end scores using the Q&H index by Turesky et al^24^

The overnight plaque indices were scored digitally using the DPIA index.^25^ Also, a significant difference (DiffM‐3.15, 95% CI [−4.61:‐1.69], *P* < .001) was observed in favour of the active ingredients triclosan or stannous fluoride. See Appendix S7 for the funnel plot.

### 3.9 Statistical heterogeneity

The percentage of variance in the meta‐analysis attributable to study heterogeneity was high for the studies assessing the Q&H index by Turesky et al^24^ index (*I* ^2^ = 78%[95% CI: 52%‐90%]; *P* < .01) and low for studies that assessed plaque digitally using the DPIA index^25^ (*I* ^2^ = 0%[95% CI: 0%‐45%]; *P* = .78).

### 3.10 Publication bias detection

The test for funnel plot asymmetry, based on rank correlation^31^ or linear regression method,^29^ was not significant (*P* = .46 and *P* = .55). Contour‐enhanced funnel plots and plots with trimfill^33^, ^68^ are presented in Appendix S8A,B. Since most of the missing studies are located in regions of high significance, publication bias is unlikely to be the underlying cause of asymmetry.^68^

A Copas selection model analysis was conducted to investigate, and attempt to correct for, selection/publication bias in the meta‐analysis.^34^, ^35^ Adjusting for selection bias, the Copas selection model estimated that the pooled adjusted standardized mean difference was −0.72(*P* < .0001; 95% CI: [−1.00;‐0.44]) and equalled the random‐effects model estimate of −0.72 (*P* < .0001; 95% CI: [−1.01; −0.42]). Although tests and funnel plots suggest that publication bias is not likely, it could not be ruled out. See Appendix S8C for the results of the Copas selection model analysis.

### 3.11 Trial sequential analysis

Appendix S10 presents the results of the trial sequential analyses (TSA) per index used. TSA of this MA showed that the effect was conclusive and reliable, and that additional data are unlikely to affect the summary effect.^41^

### 3.12 Post hoc sensitivity analysis study design

Three post hoc sensitivity analyses of the crossover trials using stannous fluoride were performed in order to confirm the robustness of the results of the MA.^69^ A within‐patient correlation of 0.5 was assumed because information of the required matched outcome data was not available.^17^, ^70^ The sensitivity analysis of the crossover trials with correlation coefficients of 0, 0.25 and 0.5 is in agreement with the results of the MA. See Appendix S11 for the results of the post hoc sensitivity analysis.

### 3.13 Additional analysis

The results of the influence or sensitivity analysis by calculating pooled estimates omitting one study at a time showed that no single study significantly influenced the pooled DiffMs. See Appendix S9 for supporting information. Concerning the inflation bias indicated the P‐curve analysis evidential value and no indication for p‐hacking, data‐mining or “selective reporting”.^42^, ^43^, ^71^, ^72^ See Appendix S8D for the P‐curve plot.

### 3.14 Clinical significance assessment

Because of the availability of sufficient data, calculation of the clinical significance or relevance was possible for three studies 58, 59, 62 with six comparisons. The final clinical relevance judgement was estimated to be clinically relevant for all but one.^59^ When the studies with stannous fluoride or triclosan were pooled, the judgement was clinically relevant for the pooled triclosan experiments and potentially clinically relevant in the case of the stannous fluoride experiments. See Appendix S12 for the results of the clinical relevance assessment.

### 3.15 Evidence profile

The data gathered are indirect as the model of interest is a research model for a proof of principle. However, the data are rather consistent and precise. Table 3 summarizes the various aspects that were used to rate the quality of the evidence as proposed by the GRADE working group.^55^

**Table 3:** Estimated evidence profile^55^ regarding the effect on the inhibition of plaque regrowth of the adjunctive use of an active dentifrice with brushing

Determinants of quality	Plaque scores	Subanalysis	
		Triclosan dentifrice	Stannous dentifrice
Study design (Appendix S2A)	8 RCTs and 2 CCTs	4 RCTs	8 RCTs and 2 CCTs
Risk of bias (Appendix S3)	Low to high	Low to moderate	Low to high
Consistency	Rather consistent	Rather consistent	Rather consistent
Directness	Generalizable	Generalizable	Generalizable
Precision (Table 1)	Precise	Precise	Precise
Reporting bias	Possible	Possible	Possible
Magnitude of the effect (Table 1)	Moderate	Moderate	Small
Strength and direction of the recommendation	Moderate‐quality evidence in favour of	Moderate‐quality evidence in favour of	Moderate‐quality evidence in favour of
**Overall recommendation**	**With the interest to inhibit overnight regrowth of plaque, consider a dentifrice product that contains either triclosan or stannous fluoride**		

Eight out of 10 studies included in this review were RCTs, which are widely considered the gold standard of study design when assessing effectiveness, assuming that they are methodologically sound.^14^ The risk of bias, caused by methodological limitations, varied among the studies from low to high. Restricting in sensitivity analyses the meta‐analyses to low or moderate risk of bias studies, the results were found to be robust. The unexplained heterogeneity in the meta‐analyses was low for the studies using the digital DPIA 25 index and moderate to considerable for the studies using the Turesky et al^24^ index, so it is reasonable to be moderate confident in the results presented. The results are consistent in different human populations in different geographic areas and are therefore considered generalizable. The intervention effects were consistent across studies, and no significant differences were found except for two comparisons. Tests and funnel plots for publication bias suggest that publication bias is not likely. The strength of a recommendation based on the quality of the evidence emerging from this review is estimated to be moderate concerning the usage of triclosan and weak concerning the usage of stannous fluoride in comparison with a regular fluoride dentifrice on the inhibition of overnight plaque regrowth.

## 4 DISCUSSION

The prevention of dental caries and periodontal diseases centres on dental plaque control. In this context, chemical agents could represent a valuable complement to mechanical plaque control.^73^ Over recent decades, studies of various agents have provided information on their efficiency in controlling or inhibiting plaque growth.^3^, ^11^, ^12^, ^14^, ^74^ However, differentiating between dentifrices in terms of their antiplaque properties also requires assessment of their ability to inhibit plaque regrowth, which is commonly measured as overnight plaque accumulation.^75^ The purpose of this systematic review (SR) was to establish to what extent a dentifrice inhibits overnight plaque regrowth.

In this SR, dentifrices containing the active ingredients stannous fluoride or triclosan were significantly more effective at inhibiting overnight plaque regrowth than regular dentifrices containing sodium fluoride. The effect of stannous fluoride was found to extend over a 24‐hour period.

In the studies with the DPIA^25^ index, no baseline scores were available, but all the participants received both interventions. Therefore, the differences as revealed in the meta‐analysis concerning the end scores demonstrated true differences in outcomes of the investigated products.

Colgate® Total® and Crest® Pro‐Health® are currently the only two dentifrices with purported antiplaque properties accepted by the ADA.^76^ Claims that chemotherapeutic products control or modify plaque may be made only if it can also be demonstrated that there is a significant effect against gingivitis.^77^, ^78^ The criteria set are that studies should show a statistically significant proportional reductions of 20% or more in indices, referring to a comparison between the active therapy and the control at the end of the study.^79^ In the present SR, the criteria of sufficient proportional reduction were only met by five out of 14 experiments. This contrasts with the studies using the digital DPIA index,^25^ where one out of five studies did not meet this criterion. The weighted mean proportional reduction for the Q&H index by Turesky et al^24^ was 12.9% and for the digital DPIA^25^ index 25.0%. Several studies indicate that this difference may find its origin in the fact that computer‐based plaque analyses are more precise, more objective and more sensitive than classic plaque indices.^80^ The Q&H index by Turesky et al^24^ is a 0‐5 integer assessment of the plaque on labial, buccal and lingual surfaces of each individual tooth.^81^ For example, if a particular tooth area is assessed as a score of one and a toothbrush removes 50% of the plaque at this site, the resultant is still one. In order for the index to be zero, the plaque must be completely removed.^25^ It is envisioned that DPIA^25^ will overcome this and other problems.^25^, ^82^ Another noteworthy difference is that DPIA^25^ performs a partial plaque measurement (the facial surfaces of 12 anterior teeth). Data from a large cross‐sectional study demonstrate that an efficient, partial mouth plaque measurement at visible sites (19% of total) was comparable to whole mouth plaque scores.^83^ This is in concordance with earlier findings from Bentley & Disney.^84^

All the studies in this SR are in some way related to the industry. Correlations between funding by industry and study outcomes are frequently observed in the literature.^85^ Studies that report positive or significant results are more likely to be published and statistically significant outcomes have higher odds of being fully reported.^86^ On the other hand, especially from renowned manufactures, the quality of the research is high because the procedures are ensured according to the criteria of good clinical practice. Moreover, several studies concluded that positive conclusions in dentifrice trials are not associated with conflict of interest or funding.^87^

Stannous fluoride and triclosan are agents known to have antimicrobial properties.^63^, ^75^, ^85^, ^88^, ^89^ The exact ingredients of the dentifrices of the included studies in this SR were not always clear. There were also differences in ppm fluoride levels in the comparisons. This may be a concern in the comparisons of dentifrices because fluoride and sodium lauryl sulphate (SLS) also have antibacterial potential.^90^ Different formulations of the same active agents may have different effect sizes.^12^ Moreover, the compositions of a dentifrice product changes in time. The current formulation of Crest® Pro‐Health® has since 2005 incorporated stabilized stannous fluoride and an ingredient for whitening benefits, sodium hexametaphosphate.^91^ In combination with zinc citrate, triclosan does not seem to be as effective as when it is formulated with Gantrez^TM^. Which effect versus a control was demonstrated to be non‐significant.^12^, ^85^ The complex compositions of dentifrices should be considered when evaluating individual ingredients.

Recently, the FDA has banned triclosan and certain other antiseptic chemicals. Products containing triclosan should now be subject to a premarket review. The US FDA, the European Commission and several national health authorities have reviewed Colgate Total with triclosan on several occasions and have approved Colgate Total as a safe and effective medicinal dentifrice up to the approved level of 0.3%. However, its effectiveness as an antimicrobial agent, the risk of antimicrobial resistance and its possible role in hormonal developmental disruption remain controversial.^92^ Beginning of 2019, Colgate has changed its formulation and has removed triclosan and has now a completely new formulation with L‐arginine and zinc.^93^

A chlorhexidine dentifrice can also be effective for plaque control. However, the inclusion of chlorhexidine in a dentifrice formulation can pose problems because chlorhexidine can be inactivated by ingredients such as flavours and anionic detergents.^94^ The side effects and tooth discoloration are an obstacle to the generalized use of chlorhexidine products and may have a negative impact on patient compliance, which limits its usefulness in daily practice.^20^, ^95^ Therefore, it was decided “a priori” not to include chlorhexidine in the present review.

### 4.1 Prediction intervals

Besides the difference in means (DiffM) and 95% confidence intervals, we also calculated 95% prediction intervals. The prediction interval can help understand the uncertainty about whether or not an intervention works.^27^ A prediction interval quantifies the dispersion of effect estimates of the interventions. In the experiments using the DPIA^25^ index, the effect of a new study will be within an interval of −5.07 and −1.24 with 95% confidence. For the studies using the Q&H index the effect of a new study will be within an interval of −0.87 and 0.21 with 95% confidence. In the latter case, the estimated probability that the true effect of the use of a dentifrice with triclosan or stannous fluoride in comparison with a regular dentifrice will be null or higher in a new study is 94%.^27^

### 4.2 Influence analysis

The leave‐one‐out method can be used in a random‐effects context to informally investigate the influence of specific studies^96^ by assessing whether these studies have a very high influence on the overall results of the meta‐analysis effect sizes. The plot highlights influential studies, as when they are left out of the analysis, the overall estimate will be notably distorted. When a sensitivity analysis shows that the overall result is not affected to a large extent, the results of the meta‐analysis give more confidence. In this review, the results of the sensitivity analyses showed that no single study significantly influenced the pooled estimates. See Appendix S9 for supporting information and plots.

### 4.3 Inflation bias analysis

Inflation bias, also known as “p‐hacking” or “selective reporting,” is assumed to occur when researchers try out several statistical analyses and/or data eligibility specifications and then selectively report those that produce significant results.^42^, ^43^, ^71^, ^72^ The P‐curve is a plot of the distribution of p‐values reported in a set of scientific studies. Comparisons between ranges of p‐values have been used to evaluate fields of research in terms of the extent to which studies have genuine evidential value, and the extent to which they suffer from bias in the selection of variables and analyses for publication.^97^ For details, see Appendix S8D.

### 4.4 Trial sequential analysis

Most systematic reviews with meta‐analyses are underpowered.^98^, ^99^ TSA is a cumulative random‐effects meta‐analysis method that estimates a “required information size” (ie required meta‐analysis sample size) using the same framework as sample size calculation for an individual RCT, but additionally accounting for heterogeneity and multiple comparisons when new RCTs are added. TSA has demonstrated the usefulness in establishing firm conclusions from a meta‐analysis.^40^, ^100^ The TSA of the Q&H index by Turesky et al^24^ index showed that the statistical evidence was conclusive.^99^, ^101^The same conclusion can be drawn with the TSA of the DPIA index^25^ for the studies with stannous fluoride. Therefore, TSA suggests that the statistical evidence of these meta‐analyses is firm for both products. The conclusion of sufficient statistical power is supported by the P‐curve in Appendix S8D‐2.

### 4.5 Post hoc sensitivity analysis study design

In a crossover trial, each participant serves as his/her own control. Between‐patient variation is removed from the treatment comparison resulting in a smaller number of patients to achieve the same statistical power. Using a crossover design results in a gain in precision in all trials.^44^ A correlation coefficient describes how similar different assessments of the interventions are within a participant.

Because the results of crossover trials are generally similar to those of parallel‐arm trials,^102^ the results of the crossover trials included in this MA were treated as parallel‐arm trials. However, treatment‐period interaction and carry‐over effects of crossover trials may jeopardize the validity of such simple inferences. Nevertheless, the results of the sensitivity analysis of the crossover trials with correlation coefficients of 0.5, 0.25 and 0 were in agreement with the results of the MA. For details, see Appendix S11.

### 4.6 Clinical significance assessment

Statistical significance analysis provides only a dichotomous answer. It may or may not be statistically significant and does not offer an indication of how important the result of the study is.^53^, ^103^ A result can be clinically relevant but might be neglected if statistical significance was not attained due to small sample sizes and high intersubject variability. Clinical relevance or clinical significance assessment indicates whether the results are meaningful or not. In the absence of normative values for the outcomes of interest, other methods must be sought for assessing clinical relevance, such as the effect size (ES),^49^ minimal important difference (MID)^50^, ^51^ and clinically relevant judgement.^51^, ^52^ Assessing and reporting the clinical relevance of the outcome in addition to the analysis of statistical significance can help to simplify the transfer of knowledge from research into practice.^53^

The clinical significance assessment as proposed^51^, ^52^ could be performed on the studies with baseline information. These were only studies that used the Q&H index by Turesky et al^24^. A mean difference between groups higher than the MID can be considered clinically relevant.^51^, ^52^ In the present review, five comparisons showed a clinically relevant result and one comparison showed no clinically relevant result. For details, see Appendix S12.

### 4.7 Limitations related to the evidence that emerges from this review

Several limitations were identified for this review.

- While there is an emerging evidence base in public health, the data in support can often be difficult to find. Indexing of journals in MEDLINE has assisted those conducting systematic reviews to more easily identify published studies. However, information technology and the processes associated with indexing are not infallible. Studies may not be correctly marked by study design which may mean they are missed in the electronic searching process.^104^
- The more resources searched, the higher the yield, and thus time and consequently the costs required to conduct a systematic review. While there is an abundance of evidence to suggest how extensive a search for randomized controlled trials (RCTs) should be, it is neither conclusive nor consistent.^105^
- Another limitation may be the use of published research papers only. The authors of this review did not have the resources to obtain data that are kept “on file” by the various dentifrice manufacturers. This is known as the “file drawer problem,” as a form of publication bias.^106^, ^107^
- Due to the focused question of this SR, no long‐term studies were involved. As a representative of home‐use, longer‐duration studies of antimicrobial properties of dentifrice are required.^108^
- The compliance of the participants to the given protocols may be considered as an important factor in the study outcomes. None of the studies mentioned that compliance was evaluated.
- Various toothbrush types were used in the studies included, and therefore, evaluation of the added benefit of the dentifrice between studies might be influenced by this diversity.
- The populations selected for studies of dental plaque assessment, in most cases, would be individuals with mild to moderate gingivitis.^79^ The question is whether it corresponds to the average person in the population. It is quite conceivable that some people with significant plaque formation benefit substantially more from a dentifrice with active ingredients than individuals do with little plaque formation.
- The clinically subjective indices are limited because inconsistent application of the index, especially in long‐term clinical trials, often leads to greater variation in the data. Also lack of sensitivity of the scale may require larger study populations to define averages.^25^
- All the included studies became available during the last two decades. However, in the majority of cases, the manner of reporting did not follow current standards, such as CONSORT 2010 and TIDieR 2014. This limitation is also reflected in the results of the risk of bias assessment. This systematic review reinforces the importance of correct and complete reporting and adherence to standards, particularly the new TIDieR checklist regarding the description and replication of interventions.^109^

## 5 CONCLUSION

This systematic review demonstrates, based on existing data, that brushing with a dentifrice with a purported active ingredient to inhibit plaque regrowth, such as stannous fluoride or triclosan, provides a significant and clinically relevant effect that surpasses the effect of a regular sodium fluoride dentifrice.

## 6 CLINICAL RELEVANCE

### 6.1 Scientific rationale for the study

Dentifrice does not provide an added effect for the mechanical removal of dental plaque. The question is whether purported active ingredients in dentifrices may inhibit dental plaque regrowth more effectively than a regular sodium dentifrice.

### 6.2 Principal findings

Active ingredients in dentifrice such as stannous fluoride or triclosan do provide an inhibiting effect on overnight plaque scores that surpass the effect of a regular sodium fluoride dentifrice.

### 6.3 Practical implications

Dentifrice does not significantly contribute to the mechanical removal of plaque but may serve as a carrier for active ingredients. The use of a dentifrice with the specific ingredient's stannous fluoride or triclosan inhibits overnight plaque regrowth more than a regular fluoride dentifrice.

## CONFLICTS OF INTEREST

The authors declare that they have no conflicts of interest.

## ETHICAL APPROVAL

Ethical approval was not required.

## References

[1] Gallagher A , Sowinski J , Bowman J , et al. The effect of brushing time and dentifrice on dental plaque removal in vivo. J Dent Hyg. 2009;83:111‐116. 19723429

[2] Pader M . Oral Hygiene Products and Practice. New York: Marcel Dekker Inc.; 1988.

[3] Davies R , Scully C , Preston AJ . Dentifrices‐an update. Med Oral Patol Oral Cir Bucal. 2010;15:e976‐982. 20711129 10.4317/medoral.15.e976

[4] Petersen PE , Ogawa H . Prevention of dental caries through the use of fluoride–the WHO approach. Community Dent Health. 2016;33:66‐68. 27352461

[5] Valkenburg C , Slot DE , Bakker EW , Van der Weijden FA . Does dentifrice use help to remove plaque? A systematic review. J Clin Periodontol. 2016;43:1050‐1058. 27513809 10.1111/jcpe.12615

[6] Paraskevas S , Rosema NA , Versteeg P , Timmerman MF , van der Velden U , van der Weijden GA . The additional effect of a dentifrice on the instant efficacy of toothbrushing: a crossover study. J Periodontol. 2007;78:1011‐1016. 17539713 10.1902/jop.2007.060339

[7] Van der Weijden GA , Slot DE . Oral hygiene in the prevention of periodontal diseases: the evidence. Periodontol 2000. 55(1):104‐123. 10.1111/j.1600-0757.2009.00337.x 21134231

[8] Addy M , Slayne MA , Wade WG . The formation and control of dental plaque–an overview. J Appl Bacteriol. 1992;73:269‐278. 1429304 10.1111/j.1365-2672.1992.tb04977.x

[9] Davies RM , Ellwood RP , Davies GM . The effectiveness of a toothpaste containing triclosan and polyvinyl‐methyl ether maleic acid copolymer in improving plaque control and gingival health: a systematic review. J Clin Periodontol. 2004;31:1029‐1033. 15560802 10.1111/j.1600-051X.2004.00614.x

[10] Hioe KP , Van der Weijden GA . The effectiveness of self‐performed mechanical plaque control with triclosan containing dentifrices. Int J Dent Hyg. 2005;3:192‐204. 16451308 10.1111/j.1601-5037.2005.00150.x

[11] Paraskevas S , Van der Weijden GA . A review of the effects of stannous fluoride on gingivitis. J Clin Periodontol. 2006;33:1‐13. 10.1111/j.1600-051X.2005.00860.x 16367849

[12] Gunsolley JC . A meta‐analysis of six‐month studies of antiplaque and antigingivitis agents. J Am Dent Assoc. 2006;137:1649‐1657. 17138709 10.14219/jada.archive.2006.0110

[13] Trombelli L , Farina R . Efficacy of triclosan‐based toothpastes in the prevention and treatment of plaque‐induced periodontal and peri‐implant diseases. Minerva Stomatol. 2013;62:71‐88. 23518778

[14] Riley P , Lamont T . Triclosan/copolymer containing toothpastes for oral health. Cochrane Database Syst Rev. 2013;12:CD010514. 10.1002/14651858.CD010514.pub2 PMC6775959 24310847

[15] White DJ , Kozak KM , Baker R , Saletta L . Plaque formation and removal assessed in vivo in a novel repeated measures imaging methodology. J Clin Dent. 2006;17:22‐26. 16838878

[16] Valkenburg C , Van der Weijden F , Slot DE . Is plaque regrowth inhibited by dentifrice? A systematic review and meta‐analysis with trial sequential analysis. Int J Dent Hyg. 2019;17:27‐38. 30169912 10.1111/idh.12364 PMC7379558

[17] Higgins JP , Green S . Cochrane Handbook for Systematic Reviews of Interventions. Chichester: Wiley‐Blackwell; 2011.

[18] Moher D , Liberati A , Tetzlaff J , Altman DG . Preferred reporting items for systematic reviews and meta‐analyses: the PRISMA statement. BMJ. 2009;339:b2535. 19622551 10.1136/bmj.b2535 PMC2714657

[19] PROSPERO . 2014 International prospective register of systematic reviews. http://www.crd.york.ac.uk/PROSPERO/. Accessed February 26, 2019.

[20] Slot DE , Berchier CE , Addy M , Van der Velden U , Van der Weijden GA . The efficacy of chlorhexidine dentifrice or gel on plaque, clinical parameters of gingival inflammation and tooth discoloration: a systematic review. Int J Dent Hyg. 2014;12:25‐35. 24034716 10.1111/idh.12050

[21] Van der Weijden F , Dell'Acqua F , Slot DE . Alveolar bone dimensional changes of post‐extraction sockets in humans: a systematic review. J Clin Periodontol. 2009;36:1048‐1058. 19929956 10.1111/j.1600-051X.2009.01482.x

[22] Higgins JPT , Deeks JJ . (editors). Chapter 7: Selecting studies and collecting data In: Higgins JPT, Green S, (editors), Cochrane Handbook for Systematic Reviews of Interventions. Chichester: Jon Wiley & Sons, 2008.

[23] Quigley GA , Hein JW . Comparative cleansing efficiency of manual and power brushing. J Am Dent Assoc. 1962;65:26‐29. 14489483 10.14219/jada.archive.1962.0184

[24] Turesky S , Gilmore ND , Glickman I . Reduced plaque formation by the chloromethyl analogue of victamine C. J Periodontol. 1970;41:41‐43. 5264376 10.1902/jop.1970.41.41.41

[25] Sagel PA , Lapujade PG , Miller JM , et al. Objective quantification of plaque using digital image analysis. Monogr Oral Sci. 2000;17:130‐143. 10949837 10.1159/000061638

[26] DerSimonian R , Laird N . Meta‐analysis in clinical trials. Control Clin Trials. 1986;7:177‐188. 3802833 10.1016/0197-2456(86)90046-2

[27] IntHout J , Ioannidis JP , Rovers MM , Goeman JJ . Plea for routinely presenting prediction intervals in meta‐analysis. BMJ open. 2016;6:e010247. 10.1136/bmjopen-2015-010247 PMC4947751 27406637

[28] Ioannidis JP , Patsopoulos NA , Evangelou E . Uncertainty in heterogeneity estimates in meta‐analyses. BMJ. 2007;335:914‐916. 17974687 10.1136/bmj.39343.408449.80 PMC2048840

[29] Egger M , Davey Smith G , Schneider M , Minder C . Bias in meta‐analysis detected by a simple, graphical test. BMJ. 1997;315:629‐634. 9310563 10.1136/bmj.315.7109.629 PMC2127453

[30] Sterne JA , Gavaghan D , Egger M . Publication and related bias in meta‐analysis: power of statistical tests and prevalence in the literature. J Clin Epidemiol. 2000;53:1119‐1129. 11106885 10.1016/s0895-4356(00)00242-0

[31] Begg CB , Mazumdar M . Operating characteristics of a rank correlation test for publication bias. Biometrics. 1994; 50(4):1088–1101. 7786990

[32] Duval S , Tweedie R . A nonparametric “trim and fill” method of accounting for publication bias in meta‐analysis. J Am Stat Assoc. 2000;95:89‐98.

[33] Peters JL , Sutton AJ , Jones DR , Abrams KR , Rushton L . Contour‐enhanced meta‐analysis funnel plots help distinguish publication bias from other causes of asymmetry. J Clin Epidemiol. 2008;61:991‐996. 18538991 10.1016/j.jclinepi.2007.11.010

[34] Schwarzer G , Carpenter J . Rücker G . Empirical evaluation suggests Copas selection model preferable to trim‐and‐fill method for selection bias in meta‐analysis. J Clin Epidemiol. 2010;63:282‐288. 19836925 10.1016/j.jclinepi.2009.05.008

[35] Schwarzer G , Carpenter J , Metasens RG . Advanced statistical methods to model and adjust for bias in meta‐analysis R‐package; 2014.

[36] DeMets DL , Lan KK . Interim analysis: the alpha spending function approach. Stat Med. 1994;13(13‐14):1341–1352. 7973215 10.1002/sim.4780131308

[37] O'Brien PC , Fleming TR . A multiple testing procedure for clinical trials. Biometrics. 1979;35:549‐556. 497341

[38] Wetterslev J , Thorlund K , Brok J , Gluud C . Trial sequential analysis may establish when firm evidence is reached in cumulative meta‐analysis. J Clin Epidemiol. 2008;61:64‐75. 18083463 10.1016/j.jclinepi.2007.03.013

[39] Brok J , Thorlund K , Wetterslev J , Gluud C . Apparently conclusive meta‐analyses may be inconclusive–Trial sequential analysis adjustment of random error risk due to repetitive testing of accumulating data in apparently conclusive neonatal meta‐analyses. Int J Epidemiol. 2009;38:287‐298. 18824466 10.1093/ije/dyn188

[40] Thorlund K , Anema A , Mills E . Interpreting meta‐analysis according to the adequacy of sample size. An example using isoniazid chemoprophylaxis for tuberculosis in purified protein derivative negative HIV‐infected individuals. Clin Epidemiol. 2010;2:57‐66. 20865104 10.2147/clep.s9242 PMC2943189

[41] Thorlund K , Engstrøm J , Wetterslev J , Imberger G , Gluud C . User Manual for Trial Sequential Analysis (TSA). Copenhagen Trial Unit, 2011. Copenhagen, Denmark: Centre for Clinical Intervention Research; 2017:1–115.

[42] Simonsohn U , Nelson LD , Simmons JP . P‐curve: a key to the file‐drawer. J Exp Psychol Gen. 2014;143:534‐547. 23855496 10.1037/a0033242

[43] Head ML , Holman L , Lanfear R , Kahn AT , Jennions MD . The extent and consequences of p‐hacking in science. PLoS Biol. 2015;13:e1002106. 25768323 10.1371/journal.pbio.1002106 PMC4359000

[44] Schwarzer G , Carpenter JR , Rücker G . Meta‐analysis with R. Berlin, Germany: Springer; 2015.

[45] Liu S , Lin Y , Liu X . Meta‐analysis of association of obstructive sleep apnea with glaucoma. J Glaucoma. 2016;25:1‐7. 26561422 10.1097/IJG.0000000000000357

[46] Baujat B , Mahe C , Pignon JP , Hill C . A graphical method for exploring heterogeneity in meta‐analyses: application to a meta‐analysis of 65 trials. Stat Med. 2002;21:2641‐2652. 12228882 10.1002/sim.1221

[47] Schwarzer G . Meta: an R package for meta‐analysis. R News. 2007;7:40‐45.

[48] Viechtbauer W . Conducting meta‐analyses in R with the metafor package. J Stat Softw. 2010;36:1‐48.

[49] Cohen J . A power primer. Psychol Bull. 1992;112:155‐159. 19565683 10.1037//0033-2909.112.1.155

[50] Guyatt GH , Osoba D , Wu AW , Wyrwich KW , Norman GR , Clinical Significance Consensus Meeting G . Methods to explain the clinical significance of health status measures. Mayo Clin Proc. 2002;77:371‐383. 11936935 10.4065/77.4.371

[51] Lemieux J , Beaton DE , Hogg‐Johnson S , Bordeleau LJ , Goodwin PJ . Three methods for minimally important difference: no relationship was found with the net proportion of patients improving. J Clin Epidemiol. 2007;60:448‐455. 17419955 10.1016/j.jclinepi.2006.08.006

[52] Musselman KE . Clinical significance testing in rehabilitation research: what, why, and how? Physical Therapy Reviews. 2007;12:287‐296.

[53] Armijo‐Olivo S , Warren S , Fuentes J , Magee DJ . Clinical relevance vs. statistical significance: using neck outcomes in patients with temporomandibular disorders as an example. Man Ther. 2011;16:563‐572. 21658987 10.1016/j.math.2011.05.006

[54] Cohen J . Statistical Power Analysis for the Behavioral Sciences, 2nd edn Hillsdale: Erlbaum Associates; 1988.

[55] GRADE . Grading of recommendations assessment, development and evaluation (short GRADE) working group; 2018 http://www.gradeworkinggroup.org/index.html. Accessed February 26, 2019.

[56] Smiley CJ , Tracy SL , Abt E , et al. Evidence‐based clinical practice guideline on the nonsurgical treatment of chronic periodontitis by means of scaling and root planing with or without adjuncts. J Am Dent Assoc. 2015;146:525‐535. 26113100 10.1016/j.adaj.2015.01.026

[57] White DJ , Kozak KM , Gibb R , Dunavent J , Klukowska M , Sagel PA . A 24‐hour dental plaque prevention study with a stannous fluoride dentifrice containing hexametaphosphate. J Contemp Dent Pract. 2006;7:1‐11. 16820802

[58] Singh S , Chaknis P , DeVizio W , Petrone M , Panagakos FS , Proskin HM . A clinical investigation of the efficacy of three commercially available dentifrices for controlling established gingivitis and supragingival plaque. J Clin Dentist. 2010;21:105. 21269039

[59] Prasad K , Anupama I , Rao N , Sreenivasan P , Subramanyam R , Kulkarni R . The effect of the dentifrice on gingivitis and dental plaque: a 6‐week clinical study in India. JIAPHD. 2015;13:4.

[60] Bellamy PG , Jhaj R , Mussett AJ , Barker ML , Klukowska M , White DJ . Comparison of a stabilized stannous fluoride/sodium hexametaphosphate dentifrice and a zinc citrate dentifrice on plaque formation measured by digital plaque imaging (DPIA) with white light illumination. J Clin Dent. 2008;19:48‐54. 18763686

[61] Bellamy PG , Boulding A , Farmer S , et al. Randomized in vivo trial evaluating plaque inhibition benefits of an advanced stannous‐containing sodium fluoride dentifrice used in conjunction with power brush technology. Int J Dent Hyg. 2014;12:89‐95. 23844867 10.1111/idh.12040 PMC4232329

[62] He T , Sun L , Li S , Ji N . The anti‐plaque efficacy of a novel stannous‐containing sodium fluoride dentifrice: a randomized and controlled clinical trial. Am J Dent. 2010;23(Spec No B):11B–16B. 21280421

[63] White DJ . Effect of a stannous fluoride dentifrice on plaque formation and removal: a digital plaque imaging study. J Clin Dent. 2007;18:21‐24. 17410952

[64] White DJ , Barker ML , Klukowska M . In vivo antiplaque efficacy of combined antimicrobial dentifrice and rinse hygiene regimens. Am J Dent. 2008;21:189‐196. 18686773

[65] Bellamy PG , Khera N , Day TN , Barker ML , Mussett AJ . A randomized clinical trial to compare plaque inhibition of a sodium fluoride/potassium nitrate dentifrice versus a stabilized stannous fluoride/sodium hexametaphosphate dentifrice. J Contemp Dent Pract. 2009;10:1‐9. 19279966

[66] Bellamy PG , Prendergast M , Strand R , et al. Can anti‐erosion dentifrices also provide effective plaque control? Int J Dent Hyg. 2011;9:223‐228. 21356021 10.1111/j.1601-5037.2010.00480.x PMC3170713

[67] Bellamy PG , Boulding A , Farmer S , Day TN , Mussett AJ , Barker ML . Clinical comparison of plaque inhibition effects of a novel stabilized stannous fluoride dentifrice and a chlorhexidine digluconate dentifrice using digital plaque imaging. J Clin Dent. 2011;22:144‐148. 22403979

[68] Sterne JA , Sutton AJ , Ioannidis JP , et al. Recommendations for examining and interpreting funnel plot asymmetry in meta‐analyses of randomised controlled trials. BMJ. 2011;343:d4002. 21784880 10.1136/bmj.d4002

[69] Elbourne DR , Altman DG , Higgins JP , Curtin F , Worthington HV , Vail A . Meta‐analyses involving cross‐over trials: methodological issues. Int J Epidemiol. 2002;31:140‐149. 11914310 10.1093/ije/31.1.140

[70] Smail‐Faugeron V , Fron‐Chabouis H , Courson F , Durieux P . Comparison of intervention effects in split‐mouth and parallel‐arm randomized controlled trials: a meta‐epidemiological study. BMC Med Res Methodol. 2014;14:64. 24886043 10.1186/1471-2288-14-64 PMC4023173

[71] Simonsohn U , Nelson LD , Simmons, JP . p‐curve and effect size: correcting for publication bias using only significant results. Perspect Psychol Sci. 2014;9:666‐681. 26186117 10.1177/1745691614553988

[72] Bishop DV , Thompson PA . Problems in using p‐curve analysis and text‐mining to detect rate of p‐hacking and evidential value. PeerJ. 2016;4:e1715. 26925335 10.7717/peerj.1715 PMC4768688

[73] Baehni PC , Takeuchi Y . Anti‐plaque agents in the prevention of biofilm‐associated oral diseases. Oral Dis. 2003;9(Suppl 1):23‐29. 12974527 10.1034/j.1601-0825.9.s1.5.x

[74] Mandel ID . Chemotherapeutic agents for controlling plaque and gingivitis. J Clin Periodontol. 1988;15:488‐498. 3053790 10.1111/j.1600-051x.1988.tb01020.x

[75] He T , Barker ML , Biesbrock AR , et al. Digital plaque imaging evaluation of a stabilized stannous fluoride dentifrice compared with a triclosan/copolymer dentifrice. Am J Dent. 2013;26:303‐306. 24640432

[76] American Dental Association (ADA) . Consumer products with the ADA Seal of Acceptance, 2018 http://www.ada.org/en/science-research/ada-seal-of-acceptance/ada-seal-products/product-category?attributes=Plaque%252fGingivitis+Control. Accessed December 20, 2018.

[77] American Dental Association (ADA) . Acceptance Program Guidelines: Chemotherapeutic Products for Control of Gingivitis. Chicago: American Dental Association (ADA); 1997:19.

[78] Pereira EM , da Silva JL , Silva FF , et al. Clinical evidence of the efficacy of a mouthwash containing propolis for the control of plaque and gingivitis: a phase II study. Evid Based Complement Alternat Med. 2011;2011:750249. 21584253 10.1155/2011/750249 PMC3092688

[79] American Dental Association (ADA) . Council on Scientific Affairs. Acceptance Program Requirements: Adjunctive Dental Therapies for the Reduction of Plaque and Gingivitis. Chicago: American Dental Association (ADA); 2011:19.

[80] Pretty I , Edgar W . Higham SM . A study to assess the efficacy of a new detergent free, whitening dentifrice in vivo using QLF planimetric analysis. Br Dent J. 2004;197:561. 15543118 10.1038/sj.bdj.4811809

[81] Fischman SL . Current status of indices of plaque. J Clin Periodontol. 1986; 13(5):371–374, 379‐380. 3013947 10.1111/j.1600-051x.1986.tb01475.x

[82] White DJ , Biesbrock AR , Klukowska M . Oral care regimens and kits. U.S. Patent Application No. 11/732,927.; 2007.

[83] Dunavent JM , Barker ML , Gerlach RW , Singh M , Papas AS . Partial versus whole mouth grading of disclosed plaque [abstract 1280]. J Dent Res. 2008;87(special Issue B).

[84] Bentley CD , Disney JA . A comparison of partial and full mouth scoring of plaque and gingivitis in oral hygiene studies. J Clin Periodontol. 1995;22:131‐135. 7775669 10.1111/j.1600-051x.1995.tb00124.x

[85] Sälzer S , Slot DE , Dörfer CE , Van der Weijden GA . Comparison of triclosan and stannous fluoride dentifrices on parameters of gingival inflammation and plaque scores: a systematic review and meta‐analysis. Int J Dent Hyg. 2015;13:1‐17. 24945592 10.1111/idh.12072

[86] Dwan K , Gamble C , Williamson PR , Kirkham JJ . Systematic review of the empirical evidence of study publication bias and outcome reporting bias ‐ an updated review. PLoS ONE. 2013;8:e66844. 23861749 10.1371/journal.pone.0066844 PMC3702538

[87] Martins CC , Riva JJ , Firmino RT , et al. Conflict of interest is not associated with positive conclusions in toothpaste trials: a systematic survey. J Clin Epidemiol. 2019;108:141–143. 30529651 10.1016/j.jclinepi.2018.11.026

[88] Kasturi R , White DJ , Lanzalaco AC , et al. Effects of nine weeks' use of a new stabilized stannous fluoride dentifrice on intrinsic plaque virulence expressed as acidogenicity and regrowth: a modified PGRM study. J Clin Dent. 1995;6(Spec No):71‐79. 8593196

[89] Peter S , Nayak D , Philip P , Bijlani N . Antiplaque and antigingivitis efficacy of toothpastes containing Triclosan and fluoride. Int Dent J. 2004;54:299‐303. 15509080 10.1111/j.1875-595x.2004.tb00002.x

[90] Otten MP , Busscher HJ , Abbas F , van der Mei HC , van Hoogmoed CG . Plaque‐left‐behind after brushing: intra‐oral reservoir for antibacterial toothpaste ingredients. Clin Oral Investig. 2012;16:1435‐1442. 10.1007/s00784-011-0648-2 PMC3443356 22160537

[91] Sensabaugh C , Sagel ME . Stannous fluoride dentifrice with sodium hexametaphosphate: review of laboratory, clinical and practice‐based data. J Dent Hyg. 2009;83:70‐78. 19470232

[92] Hogue CJC , News E . US FDA halts use of triclosan in health care antiseptics. C&EN Global Enterprise. 2018;96:15‐15.

[93] Delgado E , Garcia‐Godoy F , Montero‐Aguilar M , Mateo LR , Ryan M . A clinical investigation of a dual zinc plus arginine dentifrice in reducing established dental plaque and gingivitis over a six‐month period of product use. J Clin Dent. 2018;29:A33‐A40. 30620869

[94] Addy M , Jenkins S , Newcombe R . Studies on the effect of toothpaste rinses on plaque regrowth. (I). Influence of surfactants on chlorhexidine efficacy. J Clin Periodontol. 1989;16:380‐438. 2760250 10.1111/j.1600-051x.1989.tb00008.x

[95] Supranoto SC , Slot DE , Addy M , Van der Weijden GA . The effect of chlorhexidine dentifrice or gel versus chlorhexidine mouthwash on plaque, gingivitis, bleeding and tooth discoloration: a systematic review. Int J Dent Hyg. 2015;13:83‐92. 25059640 10.1111/idh.12078

[96] Cooper H , Hedges L , Valentine JC . Handbook of Research Synthesis and Meta‐analysis. New York: Russell Sage Foundation; 2009.

[97] Simonsohn U , Simmons JP , Nelson LD . Better P‐curves: Making P‐curve analysis more robust to errors, fraud, and ambitious P‐hacking, a Reply to Ulrich and Miller (2015). J Exp Psychol Gen. 2015;144:1146‐1152. 26595842 10.1037/xge0000104

[98] Imberger G , Thorlund K , Gluud C , Wetterslev J . False‐positive findings in Cochrane meta‐analyses with and without application of trial sequential analysis: an empirical review. BMJ Open. 2016;6:e011890. 10.1136/bmjopen-2016-011890 PMC4985805 27519923

[99] Wetterslev J , Jakobsen JC , Gluud C . Trial Sequential Analysis in systematic reviews with meta‐analysis. BMC Med Res Methodol. 2017;17:39. 28264661 10.1186/s12874-017-0315-7 PMC5397700

[100] Roshanov PS , Dennis BB , Pasic N , Garg AX , Walsh M . When is a meta‐analysis conclusive? A guide to Trial Sequential Analysis with an example of remote ischemic preconditioning for renoprotection in patients undergoing cardiac surgery. Nephrol Dial Transplant. 2017;32:ii23‐ii30. 28380638 10.1093/ndt/gfw219

[101] Brok J , Thorlund K , Gluud C , Wetterslev J . Trial sequential analysis reveals insufficient information size and potentially false positive results in many meta‐analyses. J Clin Epidemiol. 2008;61:763‐769. 18411040 10.1016/j.jclinepi.2007.10.007

[102] Lathyris DN , Trikalinos TA , Ioannidis JP . Evidence from crossover trials: empirical evaluation and comparison against parallel arm trials. Int J Epidemiol. 2007;36:422‐430. 17301102 10.1093/ije/dym001

[103] Sterne JA , Smith GDJPT . Sifting the evidence—what's wrong with significance tests? Phys Ther. 2001;81:1464‐1469. 28206639 10.1093/ptj/81.8.1464

[104] Armstrong R , Jackson N , Doyle J , Waters E , Howes F . It’s in your hands: the value of handsearching in conducting systematic reviews of public health interventions. J Public Health. 2005;27:388‐391. 10.1093/pubmed/fdi056 16311247

[105] Crumley ET , Wiebe N , Cramer K , Klassen TP , Hartling L . Which resources should be used to identify RCT/CCTs for systematic reviews: a systematic review. BMC Med Res Methodol. 2005;5:24. 16092960 10.1186/1471-2288-5-24 PMC1232852

[106] Rosenthal R . The file drawer problem and tolerance for null results. Psychol Bull. 1979;86:638.

[107] Keukenmeester RS , Slot DE , Putt MS , Van der Weijden GA . The effect of medicated, sugar‐free chewing gum on plaque and clinical parameters of gingival inflammation: a systematic review. Int J Dent Hyg. 2014;12:2‐16. 23790138 10.1111/idh.12026

[108] Slot DE , Wiggelinkhuizen L , Rosema NA , Van der Weijden GA . The efficacy of manual toothbrushes following a brushing exercise: a systematic review. Int J Dent Hyg. 2012;10:187‐197. 22672101 10.1111/j.1601-5037.2012.00557.x

[109] Hoffmann TC , Glasziou PP , Boutron I , et al. Better reporting of interventions: template for intervention description and replication (TIDieR) checklist and guide. BMJ. 2014;348:g1687. 24609605 10.1136/bmj.g1687

