# Supplementary material for: What is the effect of active ingredients in dentifrice on inhibiting the regrowth of overnight plaque? A systematic review
Source: Int J Dent Hyg. 2019 Dec 22;18(2):128–41. doi: 10.1111/idh.12423 (PMC7217014; doi:10.1111/idh.12423)
Supplement: Supplementary file 1 [file IDH-18-128-s001.pdf]

Do active ingredients in dentifrice inhibit overnight plaque regrowth?

-A Systematic Review-

C. Valkenburg*

D.E. Slot**

G.A. van der Weijden**

Online appendices

Online Supporting Information Legends

Appendix S1

Papers excluded after full text reading with details for rejection.

Appendix S2

A. Overview of the characteristics of the studies processed for data extraction.

B. The summarized demographic characteristics of the studies processed for data extraction.

Appendix S3

Methodological quality and potential risk of bias scores of the individual included studies.

Appendix S4

Mean (SD) scores for the different intervention groups, including various indices and their modifications.

Appendix S5

A descriptive summary of statistical significance of active dentifrice compared to regular dentifrice on overnight dental plaque regrowth.

Appendix S6

Forest plots of the meta-analysis; overnight morning BASELINE and END Turesky plaque scores.

Appendix S7

Forest plot of the meta-analysis; overnight morning END DPIA plaque scores.

Appendix S8

Publication bias, selection bias and inflation bias.

Appendix S9

Influence Analysis.

Appendix S10

Trial Sequential Analysis.

Appendix S11

Post-hoc sensitivity analysis of the cross-over trials included in this MA.

Appendix S12

Clinical relevance assessment.

~~**Appendix S13**~~

~~Limitations related to the evidence that emerges from this review.~~

Appendix S13

Post hoc changes to the protocol.

Appendix S14

List of abbreviations.

Appendix S1

Papers excluded after full text reading with details for rejection

**Excluded**	
**No overnight morning design**	Emling & Yankell (1988)<br>Gilbert & Williams (1987)
**Control no sodium fluoride**	Feng et al. (2016)<br>Bellamy et al. (2012)<br>Bellamy et al. (2011a)<br>Bellamy et al. (2009)<br>Bellamy et al. (2008)<br>Klukowska et al. (2008)<br>Biesbrock et al. (2007)<br>White et al. (2006)<br>White et al. (1995)<br>Saxton et al. (1988)<br>Saxton et al. (1986)
**Only microbiology data**	Hu et al. (2008)<br>Hu et al. (2010)

Appendix S2a
Overview of the characteristics of the studies processed for data extraction

**Authors (year)**	**Study design, blinding, duration, index**	**# Participants baseline (end), gender, age (mean/range), oral prophylaxis (OP), smokers**	**Groups Brands Ingredients**<br><br>**Funding**	**Regimen: use & instructions**	**Conclusions of the original authors**
**Prasad et al. (2015)**	RCT<br>Parallel<br><br>Product use: 6 wk<br><br>Blinding to product<br><br>Smoking: ?	120 (105)<br>North West Karnataka, India<br><br>♀: 16 (15%)◊<br>♂: 89 (85%)◊<br>Mean age: 28 ◊<br>Age range: 22-43 ◊<br><br>OP: no	ADF: Crest® Pro‐ Health All Good 7 Effects Toothpaste (0.54% zinc citrate, 0.64% stannous chloride and 0.33% sodium fluoride), Procter and Gamble Company, China)<br><br>ADF: Colgate® Supershakti Dental Cream (0.3% triclosan and 1000 ppm sodium monofluorophosphate),Colgate‐ Pal molive Company, India<br><br>RDF: Colgate® CIBACA Toothpaste (1000 ppm sodium monofluorophosphate),Colgate‐ Pal molive Company, India<br><br>TB: ? (soft bristled)<br><br>No funding mentioned; two out of six authors are employees of Procter and Gamble	Self-brushing, brushing demonstration technique twice per day for I min<br><br>Refrain from other oral hygiene products<br><br>Controlled prior dentifrice use: 2 wk<br>Prior dentifrice: a commercially-available fluoride dentifrice	The triclosan/sodium monofluorophosphate dentifrice demonstrated significantly less gingivitis and greater reductions of supragingival dental plaque when compared to a dentifrice containing zinc citrate/stannous chloride/sodium fluoride and to a dentifrice containing sodium monofluorophosphate.
**Bellamy et al. (2014)**	RCT<br>Cross-over (w.o. 4 days)<br><br>Product use: 2 ½ or 5 wk<br>Blinding to product<br><br>Smoking: ?	27 (27)<br><br>Employees at Procter & Gamble, Egham, UK, using a power brush<br><br>♀: 10 (37%◊)<br>♂: 17 (63%◊)<br>Mean age: 35<br>Age range: 25- 57<br><br>OP: no	ADF: blend-a-med Pro-Expert (sodium fluoride (NaF, 1450 ppm F) as the active ingredient and stannous chloride as a key excipient), Procter & Gamble, Gross Gerau, Germany).<br><br>RDF: Colgate Cavity Protection a dual fluoride source, with (1000 ppm fluoride provided by sodium monofluorophosphate and 450 ppm fluoride provided by NaF), Colgate-Palmolive Co.<br><br>TB: Oral-B Triumph 5000 power toothbrush with EB17 brush head	Self-brushing, brushing twice per day<br>30s per quadrant<br><br>a full brush head of toothpaste<br><br>Refrain from other oral hygiene products (floss users could continue to floss their back teeth only)<br><br>Controlled prior dentifrice use: ≥ 5 days<br>Prior and washout dentifrice: a 1450 ppm NaF toothpaste (Crest Decay Prevention;	A population of power toothbrush users had significantly less plaque when using a stannous-containing NaF dentifrice than when using a negative control (fluoride) dentifrice.

Authors (year)	Study design, blinding, duration, index	# Participants baseline (end), gender, age (mean/range), oral prophylaxis (OP), smokers	Groups<br>Brands<br>Ingredients<br><br>Funding	Regimen: use & instructions	Conclusions of the original authors
			plus SmartGuide, Procter & Gamble Company)<br><br>Funding by Procter and Gamble; all authors are employees of Procter and Gamble	Procter & Gamble)	
**Bellamy et al. (2011)**	RCT<br>Cross-over (w.o.4 days)<br><br>Product use: 2 ½ or 5 wk<br>Blinding to product<br>Smoking: ?	27 (27)<br><br>Conducted in UK?<br><br>♀: 15 (55.5%)<br>♂: 12 (44.5%◊)<br>Mean age: 35<br>Age range: 25-55<br><br>OP: no	ADF: blend-a-med Pro Expert ($SnCl_2$ ⁄ NaF;1450 ppm NaF formulation with $SnCl_2$ as key excipient), Procter & Gamble, Gross Gerau, Germany<br><br>RDF: Sensodyne ProNamel (NaF; 1450 ppm NaF dentifrice with potassium nitrate), GlaxoSmithKline, Istanbul, Turkey<br><br>TB: Oral-B P35 Indicator manual toothbrush; Procter & Gamble, Gross Gerau, Germany<br><br>Funding by Procter and Gamble; authors are employees of Procter and Gamble	Self-brushing, own technique;<br><br>Refrain from other oral hygiene products<br><br>Controlled prior dentifrice use: ≥ 7 days<br>Washout and prior acclimation dentifrice: Crest Decay Prevention 1450 ppm NaF	The $SnCl_2$ ⁄ NaF dentifrice provided significantly greater daytime and overnight plaque inhibition than the NaF toothpaste.
**He et al. (2010)**	RCT<br>Cross-over (w.o.7 days)<br><br>Product use: 4 days<br>Blinding to product<br>Smoking: ?	29 (28)<br><br>Conducted in Beijing, China<br>♀: 27 (93%)<br>♂: 2 (7%)<br>Mean age: 43<br>Age range: 23-57<br><br>OP: yes	ADF: Crest Pro-Health dentifrice (stannous chloride, sodium fluoride, (1450 ppm F^-)), Procter & Gamble, Guangzhou, China.<br><br>ADF: Colgate Total (0.243% sodium fluoride/ 0.30% triclosan/2% Gantrez copolymer dentifrice), Colgate-Palmolive Company, New York, NY, USA.<br><br>RDF: Crest Cavity Protection (1100 ppm sodium fluoride), Procter & Gamble, Guangzhou, China.<br><br>TB: soft American Dental Association (ADA) manual reference	Self-brushing, twice per day<br><br>Refrain from other oral hygiene products<br><br>Controlled prior dentifrice use: ? days<br>Washout and prior acclimation dentifrice: Crest Cavity Protection	A stannous-containing, non-staining sodium fluoride dentifrice with multiple cosmetic and therapeutic benefits provided superior plaque reduction relative to a negative control and comparable benefits in favor of the stannous-containing dentifrice versus a marketed anti-plaque and anti-gingivitis triclosan dentifrice.

Authors (year)	Study design, blinding, duration, index	# Participants baseline (end), gender, age (mean/range), oral prophylaxis (OP), smokers	Groups<br>Brands<br>Ingredients<br><br>Funding	Regimen: use & instructions	Conclusions of the original authors
			toothbrush, ADA, Chicago, IL, USA.<br><br>Funding by Procter and Gamble; authors are employees of Procter and Gamble.		
**Singh et al. (2010)**	RCT<br>Parallel<br><br>Product use: 6 wk<br>Blinding to product<br><br>Smoking: ?	171 (171)<br><br>Conducted in Piscataway, NJ, USA<br>♀: 103 (60%)◊<br>♂: 68 (40%)◊<br>Mean age: 40.5◊<br>Age range: 18-70◊<br><br>OP: no	ADF: Crest Pro-Health®; 0.454% stannous fluoride/sodium hexametaphosphate/zinc lactate, Procter & Gamble Company, Cincinnati, OH, USA.<br><br>ADF: Colgate Total; 0.3% triclosan/2.0% PVM/MA copolymer/0.243% sodium fluoride, Colgate-Palmolive Co., New York, NY, USA.<br><br>RDF: Crest® Cavity Protection, 0.243% sodium fluoride, Procter & Gamble Company, Cincinnati, OH, USA.<br><br>TB: ? (soft-bristled)<br><br>Funding by the Colgate-Palmolive Company; four out of six authors product-related	Self-brushing, twice per day for one minute<br><br>Refrain from other oral hygiene products<br><br>Controlled prior dentifrice use: ? days<br>Prior acclimation dentifrice: nr	The dentifrice containing triclosan, PVM/MA copolymer, and sodium fluoride provides a greater level of antiplaque and antigingivitis efficacy than does a dentifrice containing stannous fluoride, sodium hexametaphosphate, and zinc lactate.

Authors (year)	Study design, blinding, duration, index	# Participants baseline (end), gender, age (mean/range), oral prophylaxis (OP), smokers	Groups<br>Brands<br>Ingredients<br><br>Funding	Regimen: use & instructions	Conclusions of the original authors
**Bellamy et al. (2009)**	RCT<br>Cross-over (w.o.4 days)<br><br>Product use: 2 ½ wk<br><br>Blinding to product<br><br>Smoking:?	25 (25)<br><br>Panel at the London Innovation Centre (Procter & Gamble UK)<br><br>♀: 14◊ (56%)<br>♂: 11 (44%)◊<br>Mean age: 35<br>Age range: 25-57<br><br>OP: no	ADF: blend-a-med®* EXPERT GUMS PROTECTION dentifrice (0.454% stannous fluoride/sodium hexametaphosphate/sodium fluoride (SnF2/SHMP with 1450 ppm F)), Procter & Gamble, Germany<br><br>RDF: Sensodyne® ProNamel™ dentifrice (sodium fluoride/potassium nitrate (NaF/KNO3 with 1450 ppm F)), GlaxoSmithKline, Turkey)<br><br>TB: Oral-B® P35 Indicator<br><br>Funding by Procter and Gamble (not mentioned); authors are employees of Procter and Gamble	Self-brushing, own technique; twice per day<br><br>Refrain from other oral hygiene products<br><br>a full brush head of toothpaste (approximately 1g)<br><br>Controlled prior dentifrice use: 7 days<br>Washout and prior dentifrice: Crest® Decay Prevention 0.321% SnF	The SnF2/SHMP dentifrice inhibits plaque regrowth both overnight and during the day to a significantly greater degree than the NaF dentifrice.
**White (2008)**	RCT<br>Cross-over /parallel (ADF groups) (w.o.no)<br><br>Product use: 2 wk NaF and 1wk ADF<br><br>Blinding to product<br><br>Smoking: ?	17(16)<br><br>Adult employees, Mason research facility (OH, USA)<br><br>♀: NR<br>♂: NR<br>Mean age: NR<br>Age range: NR<br><br>OP: no	ADF: Crest Pro-Health®; 0.454% stannous fluoride/sodium hexametaphosphate, Procter & Gamble Company, Cincinnati, OH, USA.<br><br>ADF: Colgate Total; triclosan/PVM/MA copolymer/0.243% sodium fluoride, Colgate-Palmolive Co., New York, NY, USA.<br><br>RDF: Crest Cavity Protection Regular Dentifrice (NaF, silica abrasive, regular flavor), The Procter & Gamble Co., Cincinnati, OH, USA).<br><br>TB: Oral-B® 40, Procter & Gamble Company, Cincinnati, OH, USA.<br><br>Funding by Procter and Gamble; authors are employees of Procter and Gamble	Self-brushing, own technique; twice per day<br><br>Refrain from other oral hygiene products; subjects who flossed regularly were allowed to floss between the posterior teeth<br><br>Controlled prior dentifrice use: 1 wk<br>Prior acclimation dentifrice: Crest Cavity Protection	Stannous fluoride dentifrice was superior to triclosan dentifrice in plaque growth inhibition between toothbrushing.

Authors (year)	Study design, blinding, duration, index	# Participants baseline (end), gender, age (mean/range), oral prophylaxis (OP), smokers	Groups<br>Brands<br>Ingredients<br><br>Funding	Regimen:<br>use &<br>instructions	Conclusions of the original authors
**White (2007)**	RCT<br>Cross-over (w.o.1 wk)<br><br>Product use: 2 wk<br><br>Blinding to product<br><br>Smoking: ?	14 (14)<br>Panel Research Center (Cincinnati, USA?)<br><br>♀: ?<br>♂: ?<br>Mean age: 33<br>Age range: >18-?<br><br>OP: no	ADF; 0.454% stannous fluoride (SnF2) prototype dentifrice<br><br>RDF: Crest® Cavity Protection Regular Dentifrice (0.243% sodium fluoride (NaF) in silica base). The Procter and Gamble Company, Cincinnati, OH, USA).<br><br>TB: Oral-B® 40 brushes (The Procter and Gamble Company, Cincinnati, OH, USA<br><br>Funding by The Procter & Gamble Company; author is employee	Self-brushing, own technique; twice per day<br><br>Refrain from other oral hygiene products; subjects who flossed regularly were allowed to floss between the posterior teeth<br><br>Controlled prior dentifrice use: ≥ 1 month<br>Washout and prior dentifrice: Crest® Cavity Protection Regular Dentifrice	Use of a SnF2 dentifrice produced statistically significant reductions in dental plaque formation as compared to the similar use of a standard NaF dentifrice.
**White et al. (2006)**	CCT<br>Cross-over (w.o. 24h)<br><br>Product use: 1 wk<br><br>Blinding to product<br><br>24 hours no oral hygiene procedures<br><br>Smoking:?	16 (16)<br>Panel Research Center (Cincinnati, USA?)<br><br>♀: 10 (62,5%◊)<br>♂: 6 (37,5%◊)<br>Mean age: 33<br>Age range: 24-38<br><br>OP: no	ADF: Crest PRO-HEALTH, (0.454% stannous fluoride complemented with sodium hexametaphosphate and silica), The Procter & Gamble Co., Cincinnati, OH, USA).<br><br>RDF: Crest Cavity Protection Regular Dentifrice (NaF, silica abrasive, regular flavor), The Procter & Gamble Co., Cincinnati, OH, USA).<br><br>TB: Oral-B 40 standard brushes (Oral-B, The Procter & Gamble Company, Cincinnati, OH, USA).<br><br>Funding by Procter and Gamble; authors are employees of Procter and Gamble	Self-brushing,<br>own technique; twice per day<br><br>Refrain from other oral hygiene products; subjects who flossed regularly were allowed to floss between the posterior teeth<br><br>Controlled prior dentifrice use: 1 wk<br>Prior dentifrice: Crest Cavity Protection Regular Dentifrice<br><br>w.o. no dentifrice	The dentifrice with stabilized stannous fluoride and sodium hexametaphosphate dentifrice inhibits plaque regrowth over a 24-hour period significantly better than a standard sodium fluoride dentifrice.

Authors (year)	Study design, blinding, duration, index	# Participants baseline (end), gender, age (mean/range), oral prophylaxis (OP), smokers	Groups<br>Brands<br>Ingredients<br><br>Funding	Regimen: use & instructions	Conclusions of the original authors
**Sagel et al. (2000)**	CCT<br>Cross-over (w.o. 4 days)<br><br>Product use: 24 h<br>Blinding to product: nr<br>Smoking:?	10 (10)<br><br>Participants: ?<br>Mason (OH, USA)?<br><br><br>♀: nr<br>♂: nr<br>Mean age: nr<br>Age range: nr<br><br>OP: no	ADF: Crest plus Gum Care<br><br>RDF: Crest Regular<br><br>TB: nr<br><br>Funding ? authors are employees of Procter and Gamble	Self-brushing, own technique;<br>Two times in 24 h<br>Subjects brushed their lingual surfaces for 30 s and swished the developed slurry to the facial surfaces for 30 s.<br><br><br>No other oral hygiene was permitted for the overnight period.<br><br>Controlled prior dentifrice use: no<br>Washout and prior dentifrice: nr	In terms of treatment, Crest plus Gum Care significantly inhibited plaque regrowth better than Crest Regular

Overview of the studies processed for data extraction

DF Dentifrice
ADF Active Dentifrice
DPIA Digital Plaque Imaging Analysis
nr not reported
OP At the initial appointment, all teeth were thoroughly scaled and polished
RDF Regular (sodium fluoride) Dentifrice
TB Toothbrush
TMQHPI Turesky Modification of the Quigley-Hein Plaque Index

wk week(s)
w.o. washout period for a crossover design
? unknown/not provided
◊ calculated by the authors of this review based on the data presented in the selected paper
N.b. Procter & Gamble marketed Crest Pro-Health in China and as blend-a-med Pro-Expert in parts of Europe. {He, 2010 #5365}

Appendix S2b
The summarized demographic characteristics of the studies processed for data extraction

Study	Country	Duration	N baseline	N end	Mean age	Age range	Male (N %)	Female (N %)
Prasad et al. (2015)	India	6wk	120	105	28	22-43	89(85%)	16(15%)
Bellamy et al. (2014)	UK	5 wk	27	27	35,3	25-57	10(37%)	17(63%)
Bellamy et al. (2011)	UK?	5 wk	27	27	35	25-55	12(44.5%)	15(55.5%)
He et al. (2010)	China	4 days	29	28	43	23-57	2(6.9%)	27(93%)
Singh et al. (2010)	USA	6wk	171	171	40,5	18-70	68(40%)	103(60%)
Bellamy et al. (2009)	UK	2.5wk	25	25	35	25-57	11(44%)	14(56%)
White 2008	USA	1wk	17	16	NR	NR	NR	NR
White 2007	USA	2wk	14	14	33	>18-?	NR	NR
White 2006	USA	1wk	16	16	33	24-38	6(37.5%)	10(62.5%)
Sagel et al. (2000)	USA?	1 day	10	10	NR	NR	NR	NR
**Total**			**456**	**439**	**33.5**	**18-70**	**198(49.5%)**	**202(51.5%)**

NR Not Reported

Appendix S3
Methodological quality and potential risk of bias scores of the individual included studies

	Quality criteria \ Study	Prasad et al. (2015)	Bellamy et al. (2014)	Bellamy et al. (2011b)	He et al. (2010b)	Singh et al. (2010)	Bellamy et al. (2009a)	White (2008)	White (2007)	White et al. (2006b)	Sagel et al. (2000)
	Study design	parallel	cross-over	cross-over	cross-over	parallel	cross-over	cross-over	cross-over	cross-over	cross-over
Internal validity	Random allocation*	+	+	+	+	+	+	+	+	-	-
	Allocation concealment	?	?	?	?	?	?	?	?	?	?
	Blinded to product*	+	+	+	+	+	+	+	+	+	?
	Blinded to examiner*	+	+	+	+	+	+	?	?	-	-
	Blinding during statistical analysis	?	?	?	?	?	?	?	?	?	?
	Balanced experimental groups*	+	+	+	+	+	+	+	+	+	+
	Reported loss to follow-up*	+	+	+	+	+	+	+	+	+	+
	# (%) of drop-outs	**15(13%) ◊**	**0(0%)**	**0(0%)**	**1(3%◊)**	**0(0%)**	**0(0%)**	**1(6%)**	**0(0%)**	**0(0%)**	**0(0%)**
	Treatment identical, except for intervention*	+	+	+	+	+	+	+	+	+	+

External validity	Representative population group	**+**	**+**	**+**	**+**	**+**	**+**	**+**	**+**	**+**	**+**
	Eligibility criteria defined*	+	+	+	+	+	+	+	+	+	?
	Sample size calculation and power	**+**	**+**	**?**	**?**	**?**	**?**	**?**	**?**	**?**	**?**
	Point estimates presented for the primary outcome	**+**	**+**	**+**	**+**	**+**	**+**	**+**	**+**	**+**	**+**
	Measures of variability presented for the primary outcome	**+**	**+**	**+**	**+**	**+**	**+**	**+**	**+**	**+**	**-**
	Unit of analysis	**subject**	**subject**	**subject**	**subject**	**subject**	**subject**	**subject**	**subject**	**subject**	**subject**
	Included a per protocol analysis	**+**	**+**	**+**	**+**	**+**	**+**	**+**	**+**	**+**	**+**
	Included an intention-to-treat analysis	**-**	**-**	**?**	**-**	**-**	**-**	**-**	**-**	**-**	**-**
Clinical aspects	Validated measurement	**+**	**+**	**+**	**+**	**+**	**+**	**+**	**+**	**+**	**+**
	Calibration examiner	**?**	**+**	**+**	**+**	**?**	**+**	**?**	**?**	**?**	**?**
	Reproducibility data shown	**?**	**+**	**+**	**?**	**?**	**+**	**+**	**+**	**+**	**?**
Authors' estimated risk of bias		**low**	**low**	**low**	**low**	**low**	**low**	**moderate**	**moderate**	**high**	**high**

Each aspect of the score list was given a rating of '+' for an informative description of the item at hand and a study design meeting the quality standard, '-' for an informative description without a study design that met the quality standard and '?' for missing or insufficient information. When random allocation, defined eligibility criteria, blinding of examiners and participants, balanced experimental groups, identical treatment between groups (except for intervention) and report of follow-up were present,

the study was classified as having a low risk of bias. When one of these seven criteria was missing, the study was considered to have a moderate potential risk of bias. When two or more of these criteria were missing, the study was considered to have a high potential risk of bias, as proposed by Van der Weijden et al. (2009).

?	not specified/unclear
+	yes
-	no
*	reporting criteria for estimating the potential risk of bias
na	not applicable
◊	calculated by the authors of this review based on the data presented in the selected paper

Appendix S4

Mean (SD) scores for the different intervention groups including various indices and their modifications

Study	Index	Group	Mean (SD)		
			Baseline	End	Difference %
Bellamy et al. (2014)	DPIA	ADF (Sn)	?	7.53(5.87◊)	?
		RDF	?	11.37(5.87◊)	?
Bellamy et al. (2011)	DPIA	ADF (Sn)	?	11.49(5.77◊)	?
		RDF	?	15.52(5.72◊)	?
Bellamy et al. (2009)	DPIA	ADF (Sn)	?	12.5(8.15◊)	?
		RDF	?	16.24(8.15◊)	?
White et al. (2008)	DPIA	ADF (Sn)	13.8(6.7)	11.3(4.7)	-18,1
		ADF (Tcs)	15.5(4.3)	15.9(4.0)	2,5
		RDF (Sn-group)	?	13.8(6.7)	?
		RDF (Tcs-group)	?	15.5(4.3)	?
White et al. (2007)	DPIA	ADF (Sn)	?	10.4(4.4)	?
		RDF	?	13.8(5.5)	?
White et al. (2006)	DPIA	ADF (Sn)	?	15.2(6.87)	?
		RDF	?	18.4(5.97)	?
Sagel et al. (2000)	DPIA	ADF (Sn)	?	?	-36.8
		RDF	?	?	12.9
Prasad et al. (2015)	TMQHPI	ADF (Sn)	2.26(0.63)	1.89(0.52)	-16.4◊
		ADF (Tcs)	2.39(0.54)	1.48(0.48)	-38.1◊
		RDF	2.30(0.53)	1.83(0.52)	-20.4◊
He et al. (2010)	TMQHPI	ADF (Sn)	3.11(0.269◊)	2.65(0.215◊)	-14.8◊
		ADF (Tcs)	3.11(0.269◊)	2.74(0.215◊)	-11.9◊
		RDF	3.04(0.265◊)	2.99(0.212◊)	-1.6◊
Singh et al. (2010)	TMQHPI	ADF (Sn)	2.27(0.41)	1.77(0.46)	-22.0
		ADF (Tcs)	2.34(0.38)	1.38(0.38)	-41.0
		RDF	2.22(0.43)	2.06(0.39)	-7.2

ADF	Active Dentifrice
RDF	Regular (sodium fluoride) Dentifrice
Sn	Stannous Dentifrice
TMQHPI	Turesky Modification of the Quigley-Hein Plaque Index
Tcs	Triclosan Dentifrice
◊	calculated by the authors of this review based on the presented data in the selected paper
?	unknown/not given
♦	additional data provided by the original authors

Appendix S5
A descriptive summary of the statistical significance of an active dentifrice compared to a regular dentifrice on overnight dental plaque regrowth

id	Study	Intervention	Plaque	Comparison
I	Prasad et al. (2015) Sn	ADF (Sn)	=	RDF
II	Bellamy et al. (2014) Sn	ADF (Sn)	>	RDF
III	Bellamy et al. (2011) Sn	ADF (Sn)	>	RDF
IV	He et al. (2010) Sn	ADF (Sn)	>	RDF
V	Singh et al. (2010) Sn	ADF (Sn)	>	RDF
VI	Bellamy et al. (2009) Sn	ADF (Sn)	>	RDF
VII	White et al. (2008) Sn	ADF (Sn)	>	RDF
IIX	White et al. (2007) Sn	ADF (Sn)	>	RDF
IX	White et al. (2006) Sn	ADF (Sn)	>	RDF
X	Sagel et al. (2000) Sn	ADF (Sn)	>	RDF
	Overall positive for ADF (Sn)		9/10	
I	Prasad et al. (2015) Tcs	ADF (Tcs)	>	RDF
IV	He et al. (2010) Tcs	ADF (Tcs)	>	RDF
V	Singh et al. (2010) Tcs	ADF (Tcs)	>	RDF
VII	White et al. (2008) Tcs	ADF (Tcs)	=	RDF
	Overall positive for ADF (Tcs)		3/4	

ADF Active dentifrice (Stannous or Triclosan dentifrice)
RDF Regular (sodium fluoride) dentifrice
Sn Stannous Dentifrice
Tcs Triclosan Dentifrice
> Significant difference in favour of test group (ADF)
< Significant difference in favour of control group (RDF)
= No significant difference
□ No data available
? Inconclusive data that does not allow conclusions concerning statistical significance

Appendix S6a

Forest plot of overnight morning BASELINE plaque scores using the Turesky modification of the Q&H (1962) plaque index clinically for the experiments in which the toothbrush was used with and without an active dentifrice; *no significant difference was observed between groups*.

Study or Subgroup	Active DF Mean	Active DF SD	Active DF Total	NaF DF Mean	NaF DF SD	NaF DF Total	Weight	Mean Difference IV, Random, 95% CI
**Tcs**								
He Tcs 2010	3.11	0.27	29	3.04	0.26	14	23.8%	0.07 [−0.10; 0.24]
Singh Tcs 2010	2.34	0.38	57	2.22	0.43	28	19.6%	0.12 [−0.07; 0.31]
Prasad Tcs 2015	2.39	0.54	42	2.30	0.53	18	7.9%	0.09 [−0.20; 0.38]
**Total (random effects, 95% CI)**			**128**			**60**	**51.3%**	**0.09 [−0.02; 0.21]**
Heterogeneity: $Tau^2 = 0$; $Chi^2 = 0.15$, df = 2 (P = 0.93); $I^2 = 0\%$ [0%; 0%]								
Test for overall effect: Z = 1.56 (P = 0.118)								
**Sn**								
He Sn 2010	3.11	0.27	29	3.04	0.26	14	23.8%	0.07 [−0.10; 0.24]
Singh Sn 2010	2.27	0.41	58	2.22	0.43	28	18.8%	0.05 [−0.14; 0.24]
Prasad Sn 2015	2.26	0.63	28	2.30	0.53	18	6.0%	−0.04 [−0.38; 0.30]
**Total (random effects, 95% CI)**			**115**			**60**	**48.7%**	**0.05 [−0.07; 0.17]**
Heterogeneity: $Tau^2 = 0$; $Chi^2 = 0.32$, df = 2 (P = 0.85); $I^2 = 0\%$ [0%; 36%]								
Test for overall effect: Z = 0.80 (P = 0.422)								
**Total (random effects, 95% CI)**			**243**			**120**	**100.0%**	**0.07 [−0.01; 0.15]**
**Prediction interval**								**[−0.05; 0.19]**
Heterogeneity: $Tau^2 = 0$; $Chi^2 = 0.74$, df = 5 (P = 0.98); $I^2 = 0\%$ [0%; 0%]								
Test for overall effect: Z = 1.68 (P = 0.093)								
Test for subgroup differences: $Chi^2 = 0.26$, df = 1 (P = 0.61)								

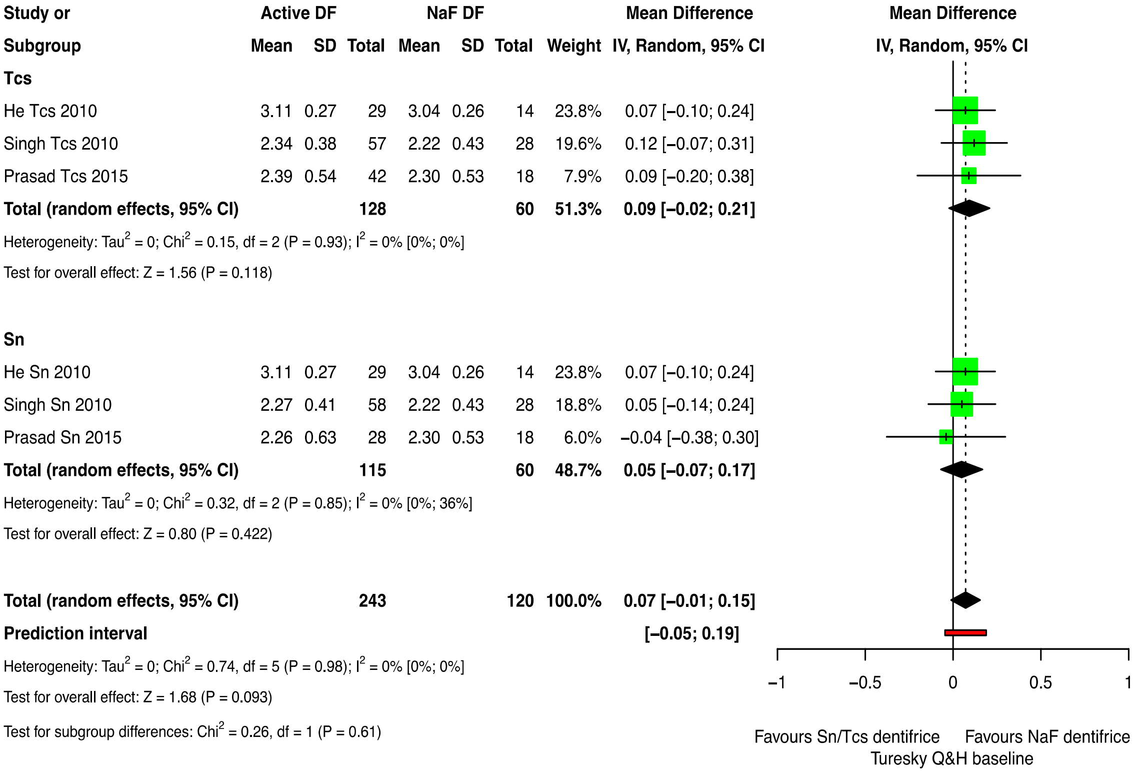

A chi-square test resulting in a p value < 0.1 was considered to be an indication of significant statistical heterogeneity. As an approximate guide for assessing the magnitude of inconsistency across studies, an I^2 statistic of 0–40% was interpreted as potentially not important, and for a statistic above 40%, moderate to considerable heterogeneity may be present.

Active Dentifrice	Stannous or Triclosan Dentifrice
Regular Dentifrice	Sodium fluoride dentifrice
Sn	Stannous Dentifrice
Tcs	Triclosan Dentifrice

Appendix S6b

Forest plot of overnight morning END plaque scores using the Turesky et al. {Turesky, 1970 #5462}modification of the Q&H (1962) plaque index clinically for the experiments in which the toothbrush was used with and without an active dentifrice; *a significant difference was observed between groups*.

Study or	Active DF			NaF DF				Mean Difference
Subgroup	Mean	SD	Total	Mean	SD	Total	Weight	IV, Random, 95% CI
**Tcs**								
He Tcs 2010	2.74	0.22	29	2.99	0.21	14	19.4%	−0.25 [−0.39; −0.11]
Singh Tcs 2010	1.38	0.38	57	2.06	0.39	28	17.8%	−0.68 [−0.85; −0.51]
Prasad Tcs 2015	1.48	0.48	42	1.83	0.52	18	13.5%	−0.35 [−0.63; −0.07]
**Total (random effects, 95% CI)**			**128**			**60**	**50.8%**	**−0.43 [−0.72; −0.13]**
Heterogeneity: Tau^2 = 0.0570; Chi^2 = 14.64, df = 2 (P < 0.01); I^2 = 86% [61%; 95%]								
Test for overall effect: Z = −2.85 (P = 0.004)								
**Sn**								
He Sn 2010	2.65	0.22	29	2.99	0.21	14	19.4%	−0.34 [−0.48; −0.20]
Singh Sn 2010	1.77	0.46	58	2.06	0.39	28	17.3%	−0.29 [−0.48; −0.10]
Prasad Sn 2015	1.89	0.52	28	1.83	0.52	18	12.5%	0.06 [−0.25; 0.37]
**Total (random effects, 95% CI)**			**115**			**60**	**49.2%**	**−0.23 [−0.42; −0.04]**
Heterogeneity: Tau^2 = 0.0173; Chi^2 = 5.44, df = 2 (P = 0.07); I^2 = 63% [0%; 89%]								
Test for overall effect: Z = −2.41 (P = 0.016)								
**Total (random effects, 95% CI)**			**243**			**120**	**100.0%**	**−0.33 [−0.49; −0.16]**
**Prediction interval**								**[−0.87; 0.21]**
Heterogeneity: Tau^2 = 0.0309; Chi^2 = 22.95, df = 5 (P < 0.01); I^2 = 78% [52%; 90%]								
Test for overall effect: Z = −3.92 (P < 0.001)								
Test for subgroup differences: Chi^2 = 1.19, df = 1 (P = 0.28)								

Mean Difference
IV, Random, 95% CI

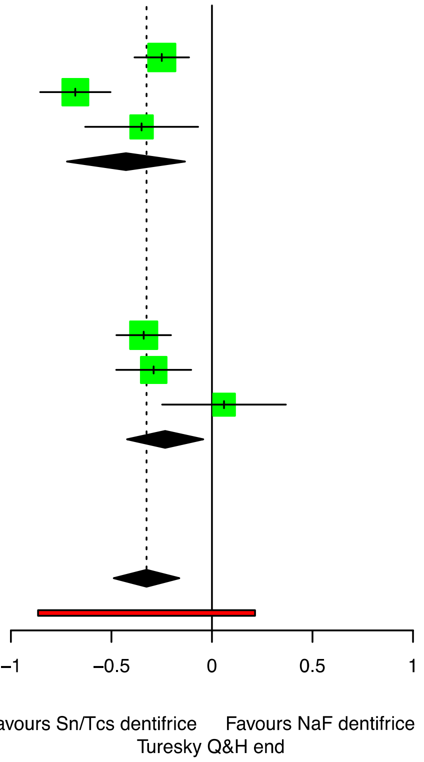

A chi-square test resulting in a p value < 0.1 was considered to be an indication of significant statistical heterogeneity. As an approximate guide for assessing the magnitude of inconsistency across studies, an I^2 statistic of 0–40% was interpreted as potentially not important, and for a statistic above 40%, moderate to considerable heterogeneity may be present.

Active Dentifrice	Stannous or Triclosan Dentifrice
Regular Dentifrice	Sodium fluoride dentifrice
Sn	Stannous Dentifrice
Tcs	Triclosan Dentifrice

Appendix S7

Forest plot of overnight morning END plaque scores using the Digital Plaque Imaging Analysis (DPIA) {Sagel, 2000 #5682}for the experiments in which the toothbrush was used with and without an active dentifrice; a significant difference was observed between groups.

Study or Subgroup	Active DF Mean	Active DF SD	Active DF Total	NaF DF Mean	NaF DF SD	NaF DF Total	Weight	Mean Difference IV, Random, 95% CI
**Sn**								
Bellamy Sn 2011	11.49	5.77	27	15.52	5.72	27	22.7%	−4.03 [−7.09; −0.97]
Bellamy Sn 2014	7.53	5.87	27	11.37	5.87	27	21.7%	−3.84 [−6.97; −0.71]
White Sn 2007	10.44	4.40	14	13.80	5.50	14	15.7%	−3.36 [−7.05; 0.33]
White Sn 2006	15.20	6.87	16	18.40	5.97	16	10.7%	−3.20 [−7.66; 1.26]
Bellamy Sn 2009	12.50	8.15	25	16.24	8.15	25	10.4%	−3.74 [−8.26; 0.78]
White Sn 2008	11.30	4.70	9	13.80	6.70	9	7.5%	−2.50 [−7.85; 2.85]
**Total (random effects, 95% CI)**			**118**			**118**	**88.7%**	**−3.60 [−5.15; −2.05]**
Heterogeneity: $Tau^2 = 0$; $Chi^2 = 0.31$, df = 5 (P = 1.00); $I^2 = 0\%$ [0%; 0%]								
Test for overall effect: Z = −4.55 (P < 0.001)								
**Tcs**								
White Tcs 2008	15.90	4.00	7	15.50	4.30	7	11.3%	0.40 [−3.95; 4.75]
**Total (random effects, 95% CI)**			**7**			**7**	**11.3%**	**0.40 [−3.95; 4.75]**
Heterogeneity: not applicable								
Test for overall effect: Z = 0.18 (P = 0.857)								
**Total (random effects, 95% CI)**			**125**			**125**	**100.0%**	**−3.15 [−4.61; −1.69]**
**Prediction interval**								**[−5.07; −1.24]**
Heterogeneity: $Tau^2 = 0$; $Chi^2 = 3.20$, df = 6 (P = 0.78); $I^2 = 0\%$ [0%; 45%]								
Test for overall effect: Z = −4.23 (P < 0.001)								
Test for subgroup differences: $Chi^2 = 2.88$, df = 1 (P = 0.09)								

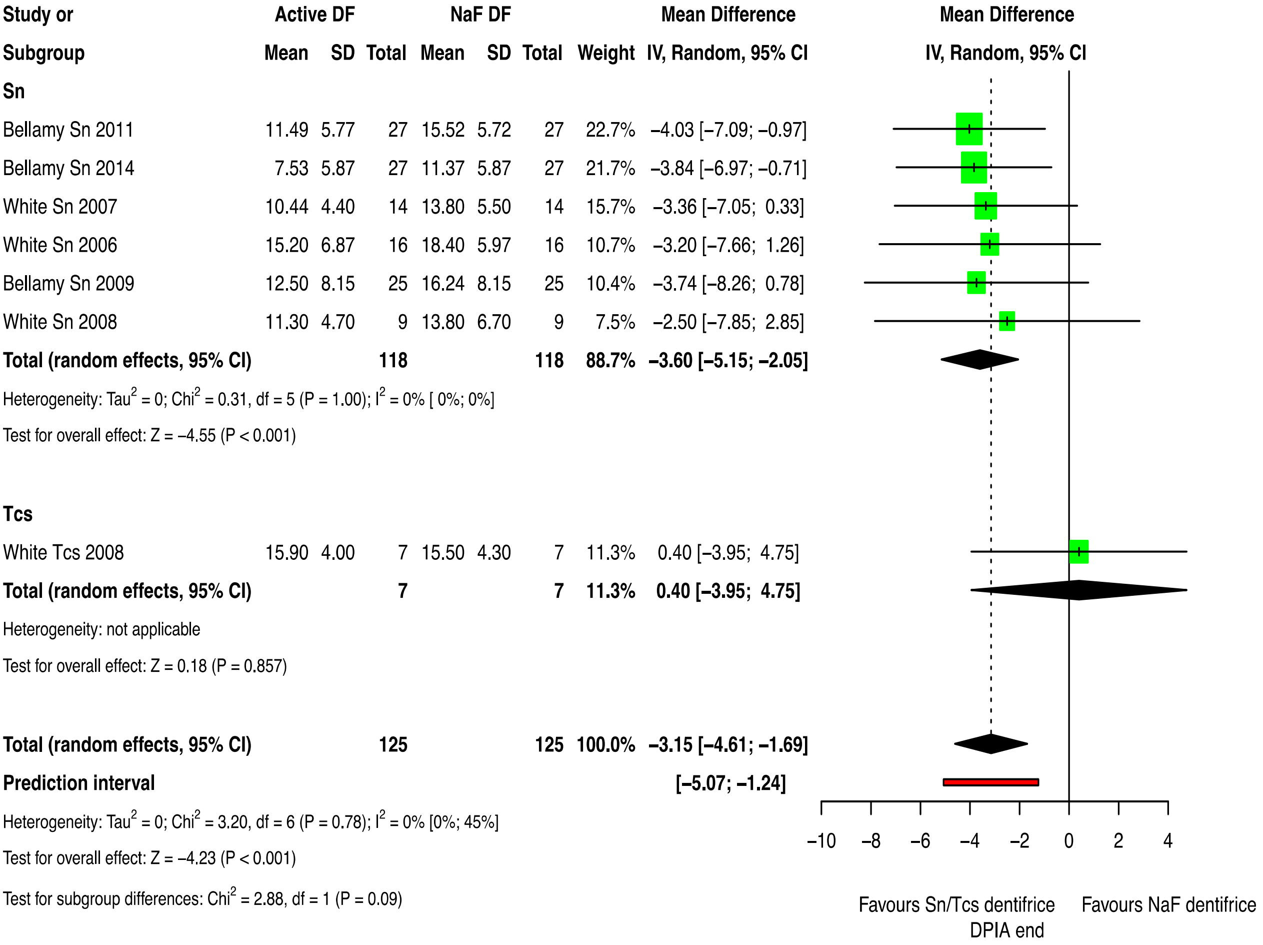

A chi-square test resulting in a p value < 0.1 was considered to be an indication of significant statistical heterogeneity. As an approximate guide for assessing the magnitude of inconsistency across studies, an I2 statistic of 0–40% was interpreted as potentially not important, and for a statistic above 40%, moderate to considerable heterogeneity may be present.

Active Dentifrice	Stannous or Triclosan Dentifrice
Regular Dentifrice	Sodium fluoride dentifrice
Sn	Stannous Dentifrice
Tcs	Triclosan Dentifrice

Appendix S8
Publication bias, selection bias, inflation bias

Although <10 publications were included, the meta-analysis was based on 13 comparisons. By pooling all comparisons using standardized mean differences (SMDs), testing of publication bias could be performed.

Appendix S8a

Contour-enhanced funnel plot of the meta-analysis showing END plaque scores of the standardized mean differences analysing the Turesky et al. (1970) {Turesky, 1970 #5462}modification of the Quigley and Hein (1962) Plaque Index and the Digital Plaque Imaging Analysis (DPIA) {Sagel, 2000 #5682}for the experiments in which the toothbrush was used with and without an active dentifrice.
Egger's test and Begg and Mazumdar test shows a non-significant p-value (P= 0.55 and P=0.46).

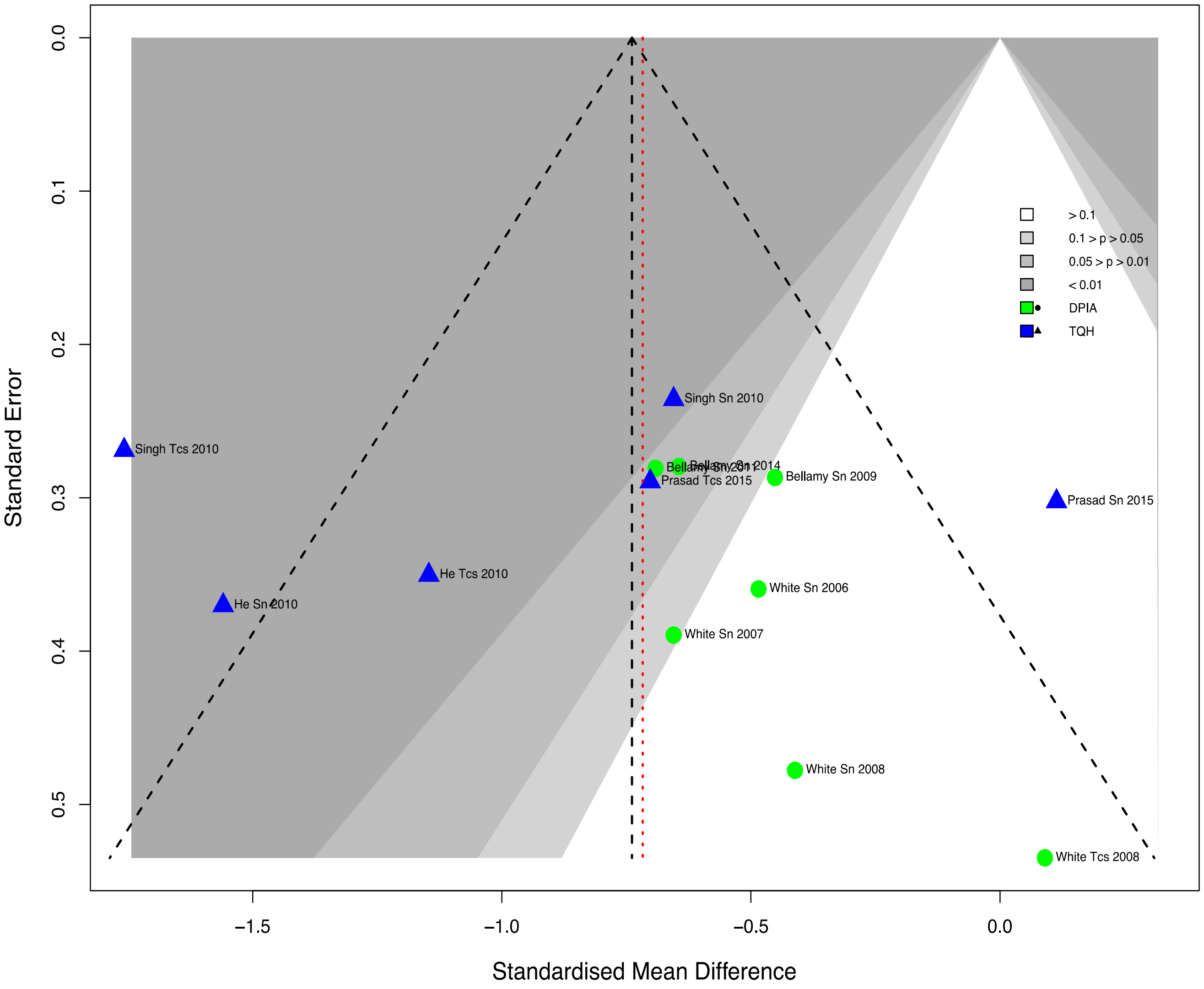

Funnel plots allow making a visual assessment of whether small-study effects may be present in a meta-analysis. A test for funnel plot asymmetry (sometimes referred to as a test for small study effects) examines whether the association between

estimated intervention effects and a measure of study size is greater than might be expected to occur by change. These tests typically have low power, so even when a test does not provide evidence of asymmetry, bias cannot be excluded {Sterne, 2011 #5859}. The counter-enhanced funnel plot is an enhancement to the usual funnel plot proposed to allow considering the statistical significance of study estimates {Peters, 2008 #6004}.
The above funnel is centered at the model estimate. Color of line represent random effects estimate. Contour lines representing well established levels of statistical significance are added to the funnel plot to indicate regions where a test of treatment effect is significant. Contour lines indicating conventional milestones in levels of statistical significance (e.g., <0.01, <0.05, <0.1) are added. P-values correspond to a trial's treatment effect {Schwarzer, 2015 #6005}. The unshaded (i.e., white) region in the middle corresponds to non-significant results, the medium gray region to significant results at the 5% level and the dark gray region to the 1% level. Visually, shows the funnel plot asymmetry. However, Egger's test and Begg and Mazumdar test the for asymmetry are not significant (P=0.55 and P=0.46), indicating a publication bias mechanism is not a major cause for concern here {Begg, 1994 #6198;Sterne, 2011 #5859}

Appendix S8b
Contour-enhanced funnel plot with trimfill of the meta-analysis showing END plaque scores of the standardized mean differences analysing the Turesky et al. (1970) {Turesky, 1970 #5462}modification of the Quigley and Hein (1962) Plaque Index and the Digital Plaque Imaging Analysis (DPIA) {Sagel, 2000 #5682}for the experiments in which the toothbrush was used with and without an active dentifrice. Egger's test and Begg and Mazumdar test shows a non-significant p-value (P= 0.90 and P=0.89).

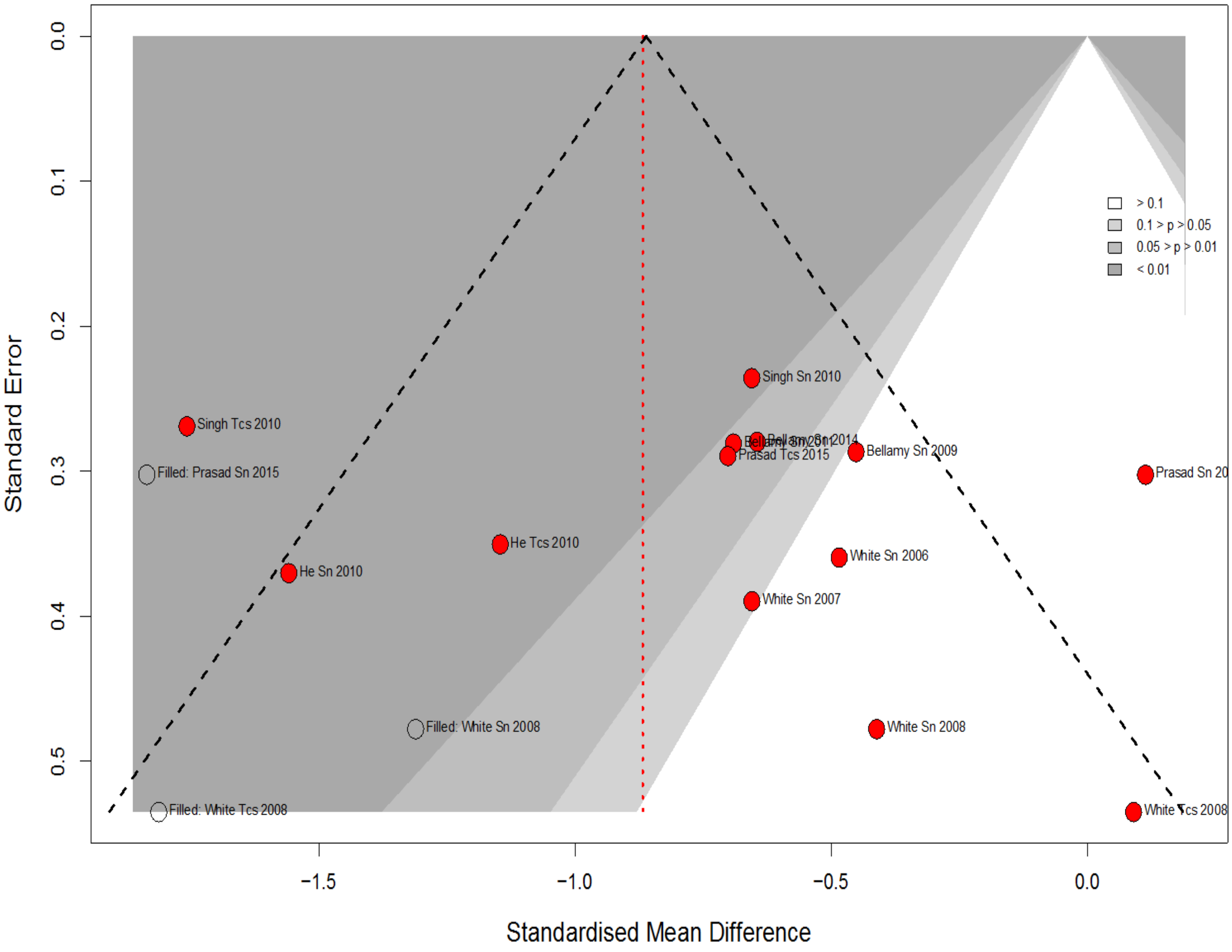

The open circles are missing comparisons filled in by a Trim-and-Filled Method. The basic idea of the trim-and-fill method is to add studies to the funnel plot until it becomes symmetric {Schwarzer, 2015 #6005}. The counter-funnel plot could be used naturally in conjunction with the trim-and-fill method because the latter informs the likely location of missing studies {Peters, 2008 #6004}.
Egger's test and Begg and Mazumdar test shows no significant p-value (P=0.90 and P=0.89) leading to acceptance of the null hypothesis of symmetry in the funnel plot {Schwarzer, 2015 #6005}.

Appendix S8c

Copas Selection Model of the meta-analysis showing END plaque scores of the standardized mean differences analysing the Turesky et al. (1970) {Turesky, 1970 #5462}modification of the Quigley and Hein (1962) Plaque Index and the Digital Plaque Imaging Analysis (DPIA) {Sagel, 2000 #5682}for the experiments in which the toothbrush was used with and without an active dentifrice. The Copas selection model analysis suggests that after accounting for selection bias and/or other small study effects, that the treatment is effective.

Model	SMD [95% CI]	P-value for hypothesis of overall treatment effect	P-value for hypothesis that no selection remains unexplained	Approximate number of unpublished studies suggested by model
Copas selection	-0.72 [-1.00; -0.44]	< 0.0001	0.3671	0
Random Effects	-0.72 [-1.01; -0.42]	< 0.0001	NA	NA
Fixed Effect	-0.74 [-0.91; -0.57]	< 0.0001	NA	NA
Prediction interval	[-1.71; 0.27]	NA	NA	NA

The Copas selection model simultaneously models the outcome and selection in which the chance of publication of a study is inversely proportional to the standard error of its outcome. The stronger the correlation , the greater the chance that only the more extreme treatment effects are selected for publication and observed by others {Schwarzer, 2010 #6208}.

ONLINE Appendix S8d Inflation bias

S8d-1) Inflation bias, also known as “p-hacking” or “selective reporting,” is assumed to occur when researchers try out several statistical analyses and/or data eligibility specifications and then selectively report those that produce significant results; researchers recording many response variables and decide which to report post analysis, deciding whether to include or drop outliers post analyses, excluding, combining, or splitting treatment groups post analysis, and stopping data exploration if an analysis yields a significant p-value[{Simonsohn, 2014 #6224;Simonsohn, 2014 #6226;Head, 2015 #6227;Bishop, 2016 #6228}]. The P-curve is a plot of the distribution of p-values reported in a set of scientific studies. Comparisons between ranges of p-values have been used to evaluate fields of research in terms of the extent to which studies have genuine evidential value, and the extent to which they suffer from bias in the selection of variables and analyses for publication, p-hacking {Simonsohn, 2015 #6225}.

P-curve analysis of the overall set of effects. Figure taken from output of the “p-curve app” (version 4.06) available at www. P-curve.com [a].

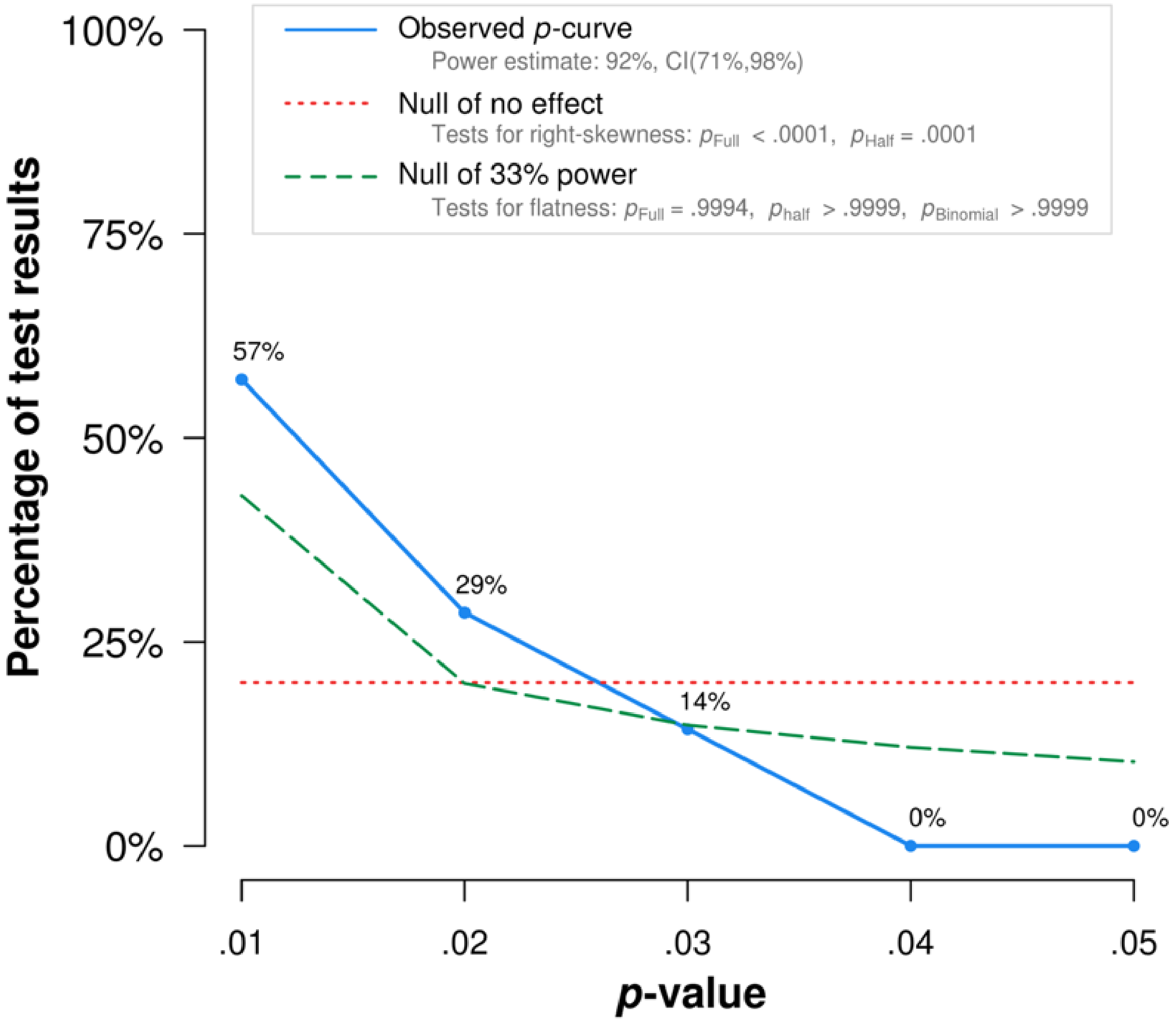

Note: The observed *p*-curve includes 7 statistically significant ($p < .05$) results, of which 7 are $p < .025$. There were 6 additional results entered but excluded from *p*-curve because they were $p > .05$.

P-curve analysis combines the half and full p-curve to make inferences about evidential value. Here both conditions are met, indicating evidential value. The P-curve plot does not indicate whether evidential value is inadequate nor absent. There is no indication for p-hacking.

S8d-2) Estimating underlying statistical power

With P-curve, the actual underlying power can be estimated, corrected for publication bias.

Estimating underlying statistical power

(Plot should be V shaped, or a smooth line to 99%)

.

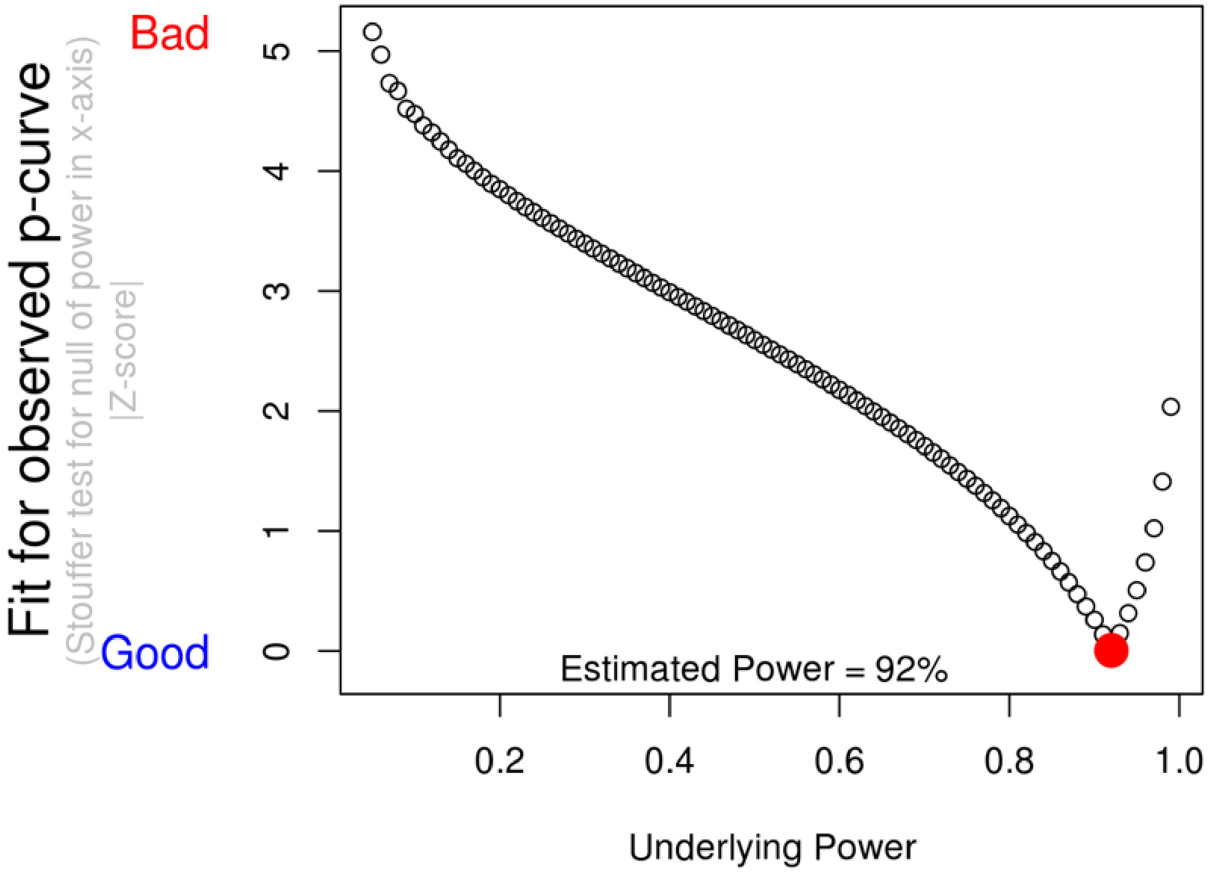

The estimated power is **92%.**

The plot estimates the power behind the data, meaning if there are sufficient studies with sufficient participants to find a true effect if it exists. A conventional threshold for optimal power is 80%, but P-curve can even assess evidential value if studies are underpowered [b].

Additional references:

a www. P-curve.com

b https://bookdown.org/MathiasHarrer/Doing_Meta_Analysis_in_R/

ONLINE Appendix S8e
Additional specialised analyses

S8e-1A) Rain forest plot of the meta-analysis showing END plaque scores of the standardized mean differences analysing the Turesky et al. (1970) {Turesky, 1970 #5462}modification of the Quigley and Hein (1962) Plaque Index (TQH) and the Digital Plaque Imaging Analysis (DPIA) {Sagel, 2000 #5682}for the experiments in which the toothbrush was used with and without an active dentifrice. The calculations were made using the metaviz package [a,b].

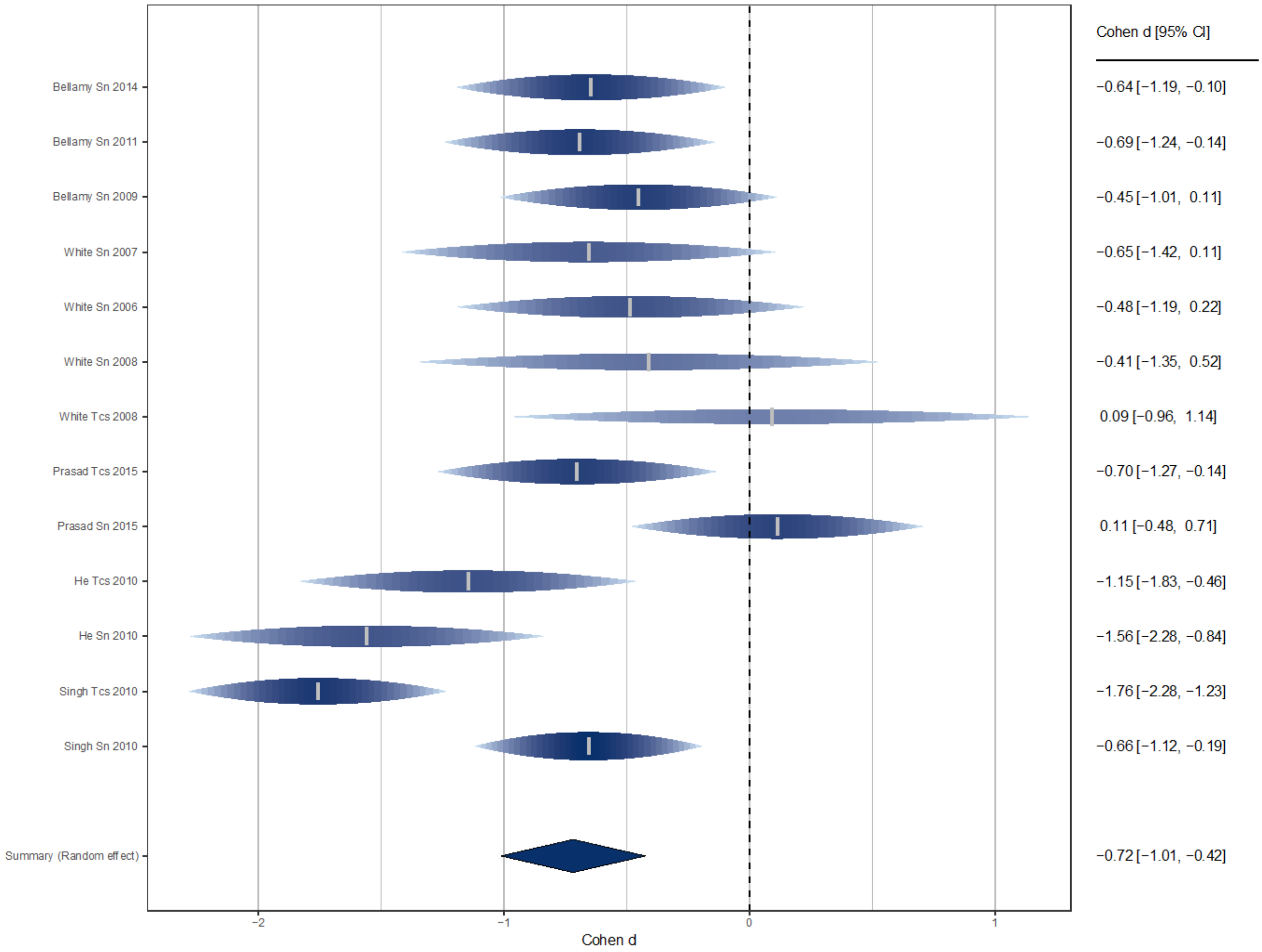

Rainforest plots have been proposed to overcome potentially misleading aspects of conventional forest plots; small studies are visually overemphasized by long confidence interval lines, which is misleading; point estimates of large studies are difficult to discern because of the large box representing the precision of the estimate within studies; confidence intervals depicted by lines might incorrectly convey the impression that all points within the interval are equally likely.
In rainforest plots, the confidence interval is marked by a horizontal white line, and its width corresponds to the width of the raindrop. In addition, the uncertainty is represented by both the height of the raindrop and the shading [c].

S8e-1B) Thick forest plot of the meta-analysis showing END plaque scores of the standardized mean differences analysing the Turesky et al. (1970) modification of the Quigley and Hein (1962) Plaque Index (TQH) and the Digital Plaque Imaging Analysis (DPIA) {Sagel, 2000 #5682}for the experiments in which the toothbrush was used with and without an active dentifrice. The calculations were made using the metaviz package [a,b].

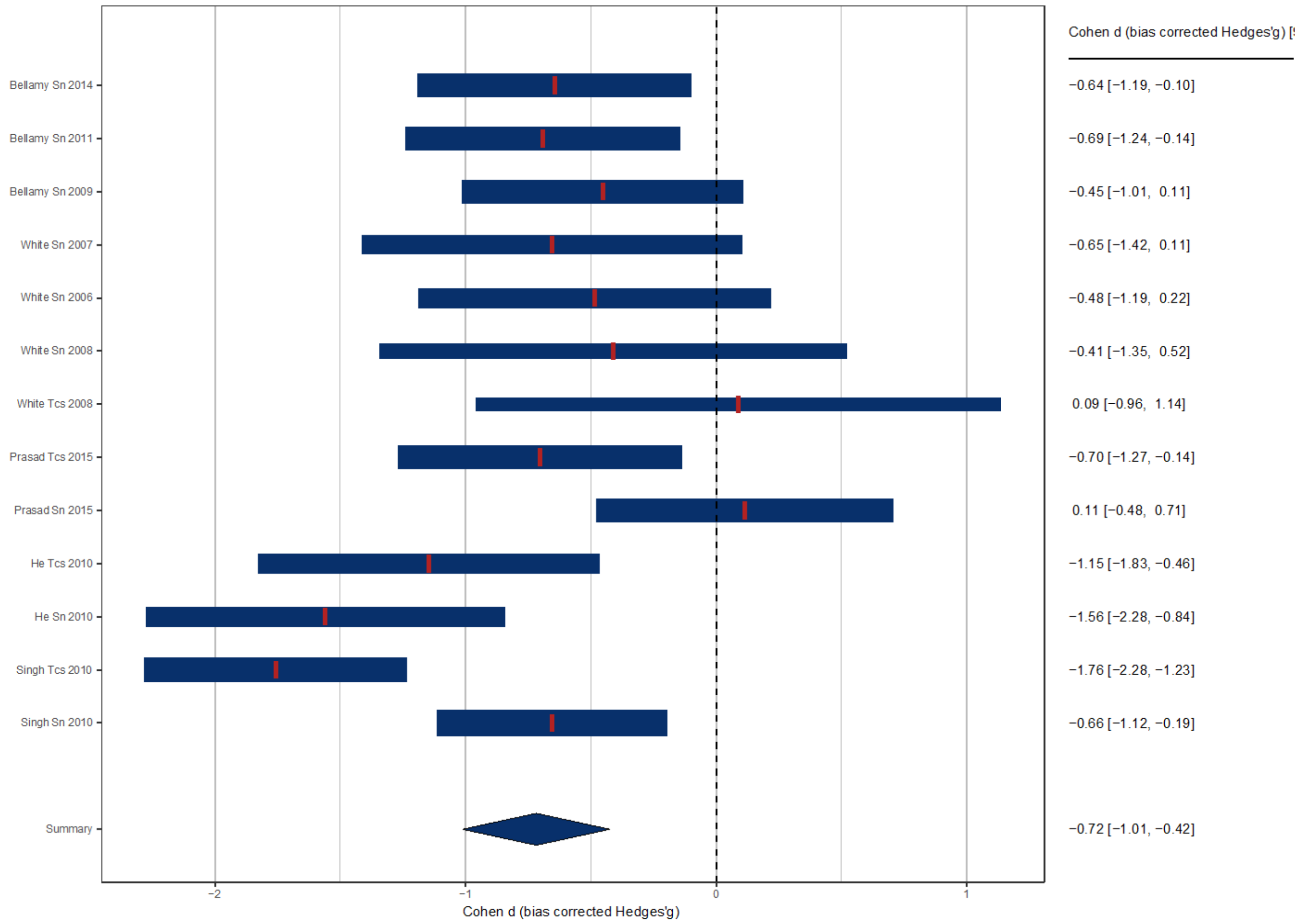

Thick forest plots have the following advantages, as compared to classic forest plots:
1. Using the height of bars proportional to the (relative) meta-analytic weight causes small studies (with wide confidence intervals and less weight in the meta-analysis) to be visually less dominant.
2. In classic forest plots, it is often hard to depict the magnitude of point estimates to a reasonable degree of accuracy, especially for studies with large meta-analytic weights and correspondingly large plotting symbols (commonly squares). Specific symbols within the thick forest plot improve the visualization of study point estimates [d].

S8e-1C) Thick forest plot of the meta-analysis. The clinically significant assessment as proposed could only be performed on the studies with baseline information. Analysis showing END plaque scores of the standardized mean differences analysing the Turesky et al. (1970) {Turesky, 1970 #5462}modification of the Quigley and Hein (1962) Plaque Index (TQH) and the Digital Plaque Imaging Analysis (DPIA) {Sagel, 2000 #5682}for the experiments in which the toothbrush was used with and without an active dentifrice. The subgroups are based on the index used (TQH and DPIA). The calculations were made using the metaviz package [a,b].

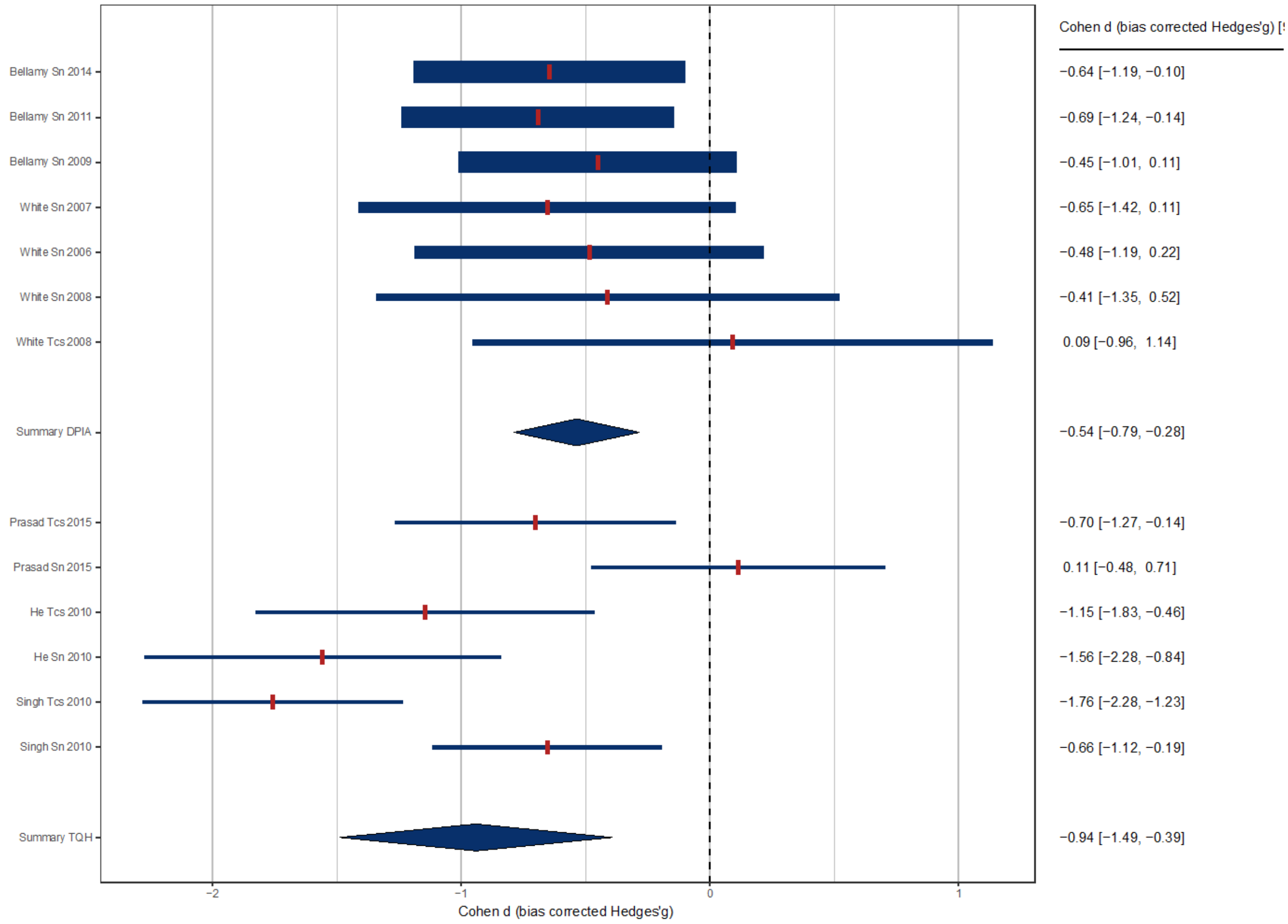

Note that for subgroup analysis the height of each error bar is scaled by the weight of each study within the subgroup divided by the sum of the weights of all studies irrespective of subgroup. Therefore, with subgroups present, the overall impression of error bar heights within a given subgroup compared to other subgroups conveys information about the relative precision of the meta-analytic estimate within the subgroup.

S8f-1A) Outlier detection

Outlier probability. Testing the presence of outliers using a parametric bootstrap with the R package metaplus [e]. This package is an additional specialised analyses and can test for the presence of outliers and compare the results of the robust and standard methods for both meta-analysis and meta-regression.

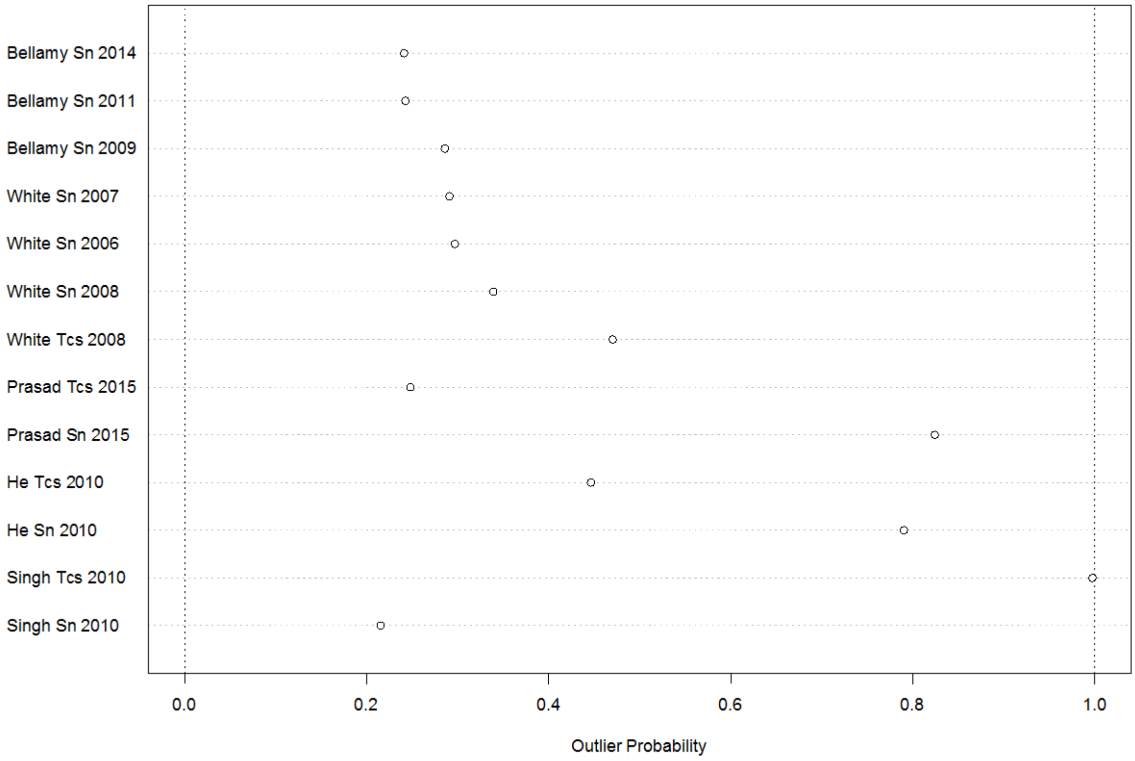

Plot of the outlier probabilities for the included studies from the robust mixture random effect model.

The plot demonstrates clearly that the study "Singh Tcs 2010" has a posterior probability of nearly 1.0 of being an outlier. This conclusion is endorsed by the output from the robust mixture model (see below). For standard studies the estimated random effect variance is zero, indicating that only the outlier studies are contributing to the heterogeneity.

	Est.	95% ci.lb	95% ci.ub	p-value
muhat	-0.6565	-0.9515	-0.4112	0.00011
tau2 (variance of the random effect for standard studies)	0.0000			
tau2out (variance for outlier studies)	0.3903			
Outlier prob.	0.4377			

S8f-1B) Forest plot for the included studies (standardized mean difference measures) with the robust meta-analysis models (t-distribution random effect and mixture of normal random effects model)

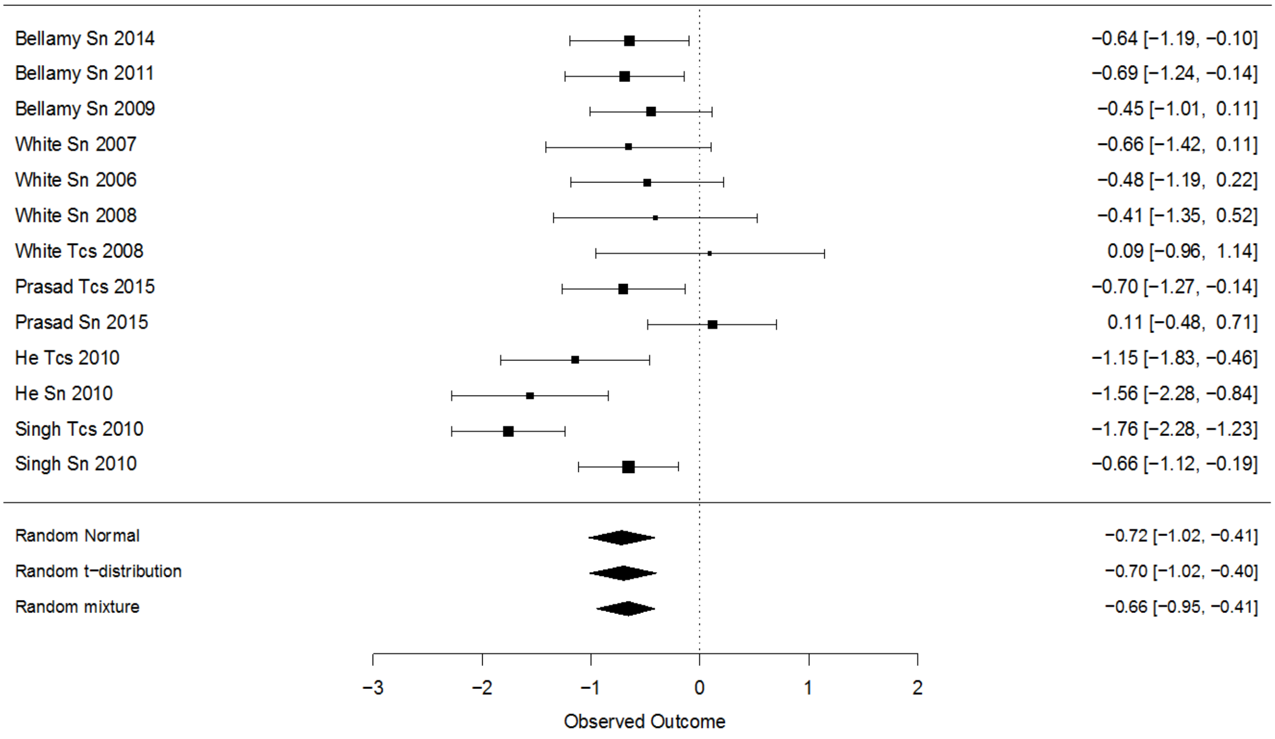

Forest plot for the included studies (standardized mean difference measures) with summaries of the standard normal effect model and the robust meta-analysis models (t-distribution random effect and mixture of normal random effects model).

The purpose of the metaplus package [e] is to fit the two robust models with random effects based on the t-distribution and the mixture of normal, as well as the standard normal random effects model.
A forest plot with the results of all three models is generated, where it can be noted that "Singh Tcs 2010" has an unusually high value. The effect of the robust models is to down-weight the "Singh Tcs 2010" study, which has the consequence of both reducing the overall effect estimate and its standard error.
Where there is little difference between the fits the mixture distribution may be preferred as it allows identification of the outlier studies [e].

S8f-2) Outlier detection in meta-analysis with altmeta package [f]

Outliers frequently appear in meta-analysis. It is recommended to study alternative approaches to robustly estimating overall effects size in the presence of outliers [f]. It is possible that different outlier detection methods identify different outliers [f]. Outliers may cause heterogeneity to be overestimated and thus affect procedures to detect them [f]. However, even if outliers are identified, there is no consensus in the statistical literature on what to do about them unless these studies are evidently erroneous [g]. No widely accepted guidelines exist for handling outliers, including the area of meta-analysis [f].

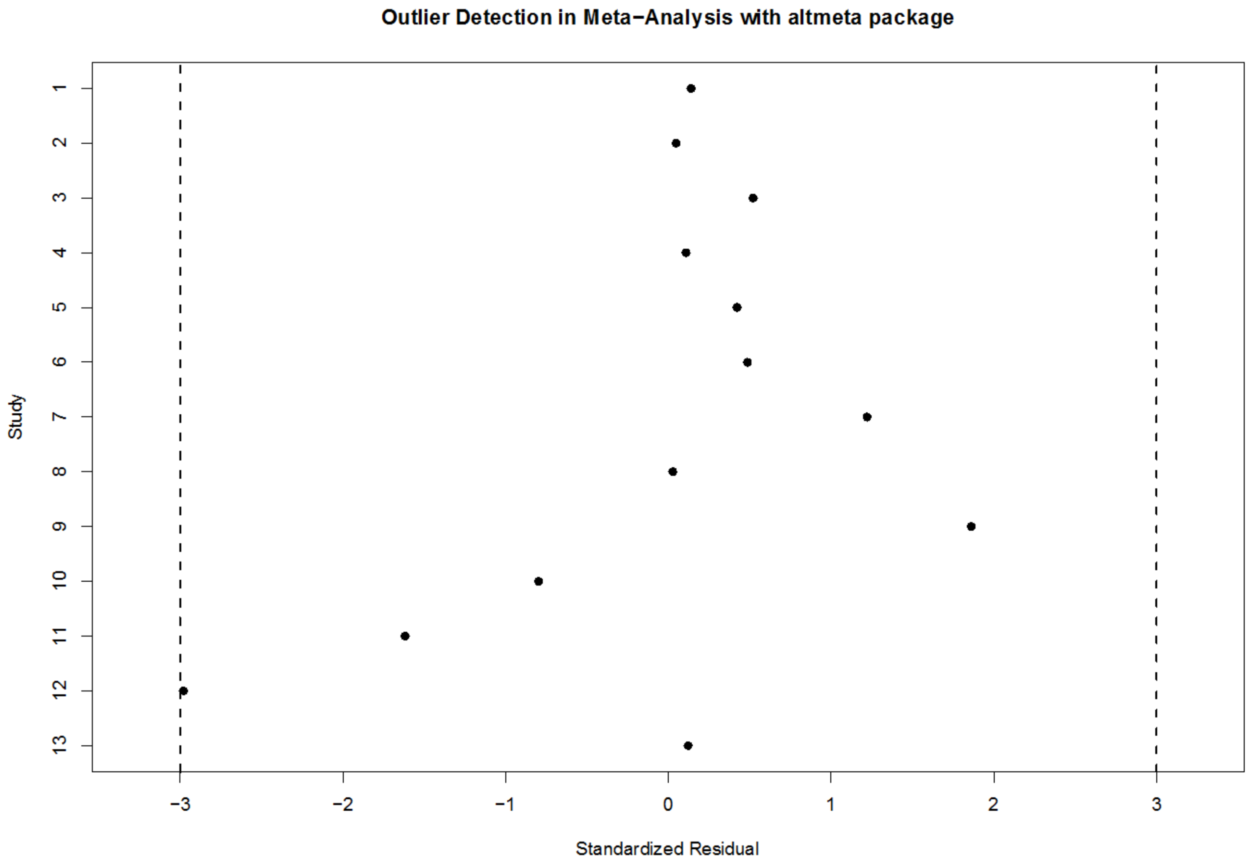

Standardized residual plot of the included studies in the meta-analysis.

A study is considered as an outlier if its standardized residual is greater than 3 in absolute magnitude [b]. Therefore, it can be concluded that the study “Singh Tcs 2010” is an outlier.

Appendix S9 Influence or Sensitivity Analysis

S9A-1) Influence analysis of studies using the Turesky et al. (1970) {Turesky, 1970 #5462}modification of the Q&H (1962) plaque index clinically for the experiments in which the toothbrush was used with and without an active dentifrice.

```
Influential analysis (Random effects model)

                                MD             95%-CI  p-value
tau^2    I^2
Omitting Singh Tcs 2010    -0.2671 [-0.3683; -0.1658] < 0.0001
0.0041  31.7%
Omitting Prasad Tcs 2015   -0.3203 [-0.5070; -0.1335]   0.0008
0.0361  82.6%
Omitting He Sn 2010        -0.3174 [-0.5336; -0.1012]   0.0040
0.0483  82.6%
Omitting Singh Sn 2010     -0.3303 [-0.5292; -0.1315]   0.0011
0.0402  82.3%
Omitting He Tcs 2010       -0.3399 [-0.5440; -0.1358]   0.0011
0.0419  80.4%
Omitting Prasad Sn 2015    -0.3805 [-0.5347; -0.2262] < 0.0001
0.0224  74.9%

Pooled estimate            -0.3260 [-0.4891; -0.1628] < 0.0001
0.0309  78.2%

Details on meta-analytical method:
- Inverse variance method
- DerSimonian-Laird estimator for tau^2
```

S9A-2) Baujat Plot {Baujat, 2002 #6195} of overnight morning END plaque scores using the Turesky {Turesky, 1970 #5462} modification of the Q&H (1962) plaque index clinically for the experiments in which the toothbrush was used with and without an active dentifrice

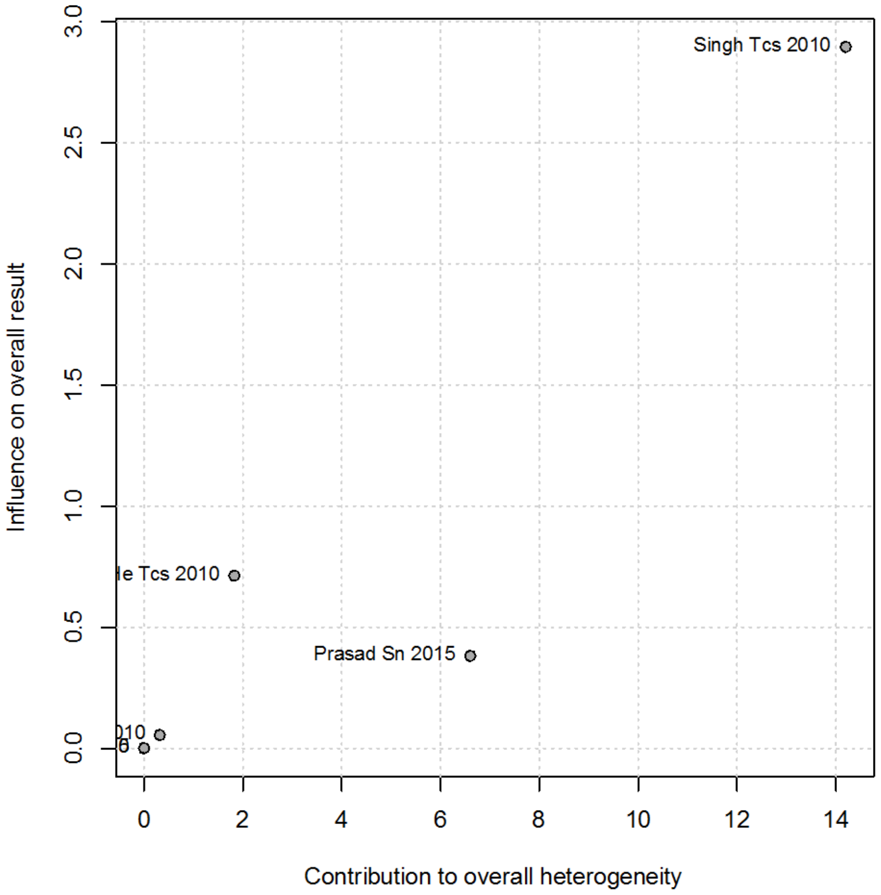

Whenever the results of trials in a meta-analysis are heterogeneous, the interpretation of the overall result may be difficult, especially if the differences between trials are not readily ascribable to variation in population characteristics {Baujat, 2002 #6195}. The Baujat Plot {Baujat, 2002 #6195} is a diagnostic plot to detect studies overly contributing to the heterogeneity of a meta-analysis. The most heterogeneous and influential trials appear in the upper right area of the graph {Baujat, 2002 #6195}. The plot shows that the experiment Singh Tcs 2010 contribute much to the overall heterogeneity and is an influence trial in the overall pooled effect of the meta-analysis.

S9A-3) Galbraith Plot of overnight morning END plaque scores using the Turesky et al. (1970) {Turesky, 1970 #5462}modification of the Q&H (1962) plaque index clinically for the experiments in which the toothbrush was used with and without an active dentifrice

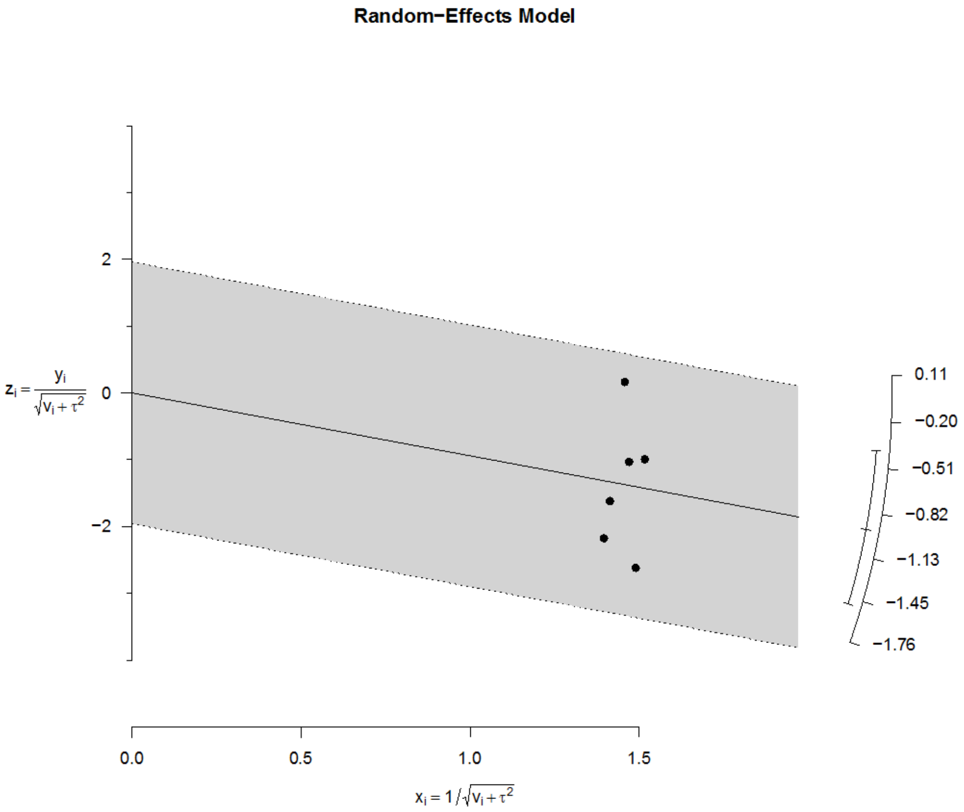

Galbraith's radial plot applied to data from END plaque scores. Each trial is plotted as a dot. Heterogeneous trials are visualized beyond the lines representing the approximated 95 per cent confidence limits.
The plot does not show specific trials as "problematic" sources of heterogeneity.

S9A-4a) Leave-One-Out-Analyses ordered by heterogeneity for the experiments using the Turesky et al. (1970) {Turesky, 1970 #5462}modification of the Q&H (1962) plaque index

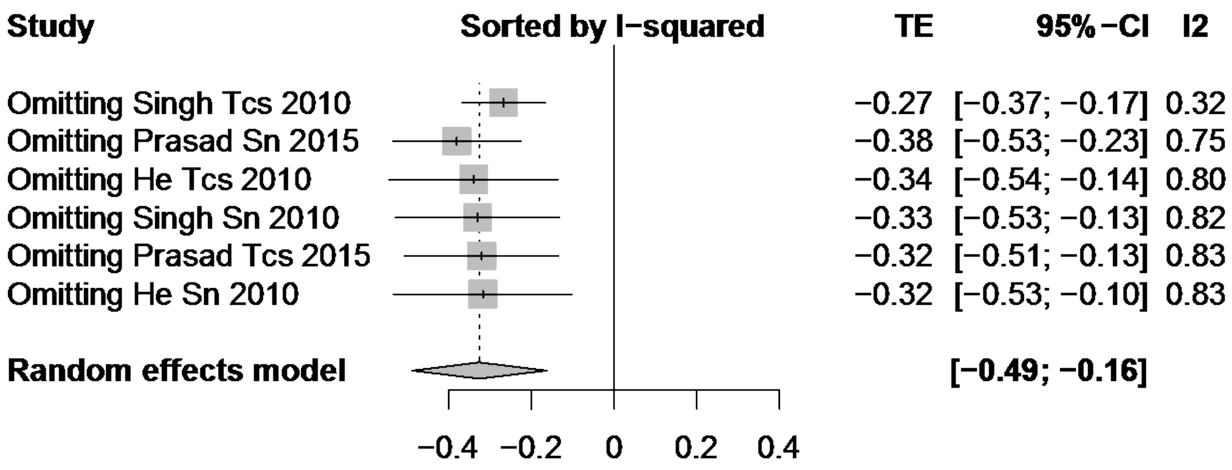

The plot is ordered by heterogeneity (low to high), as measured by I^2. From the plot it can be deduced that the lowest I^2 heterogeneity is reached by omitting the experiment Sing Tcs 2010.

S9A-4b) Leave-One-Out-Analyses ordered by Effect size for the experiments using the Turesky et al. {Turesky, 1970 #5462}modification of the Q&H (1962) plaque index**.**

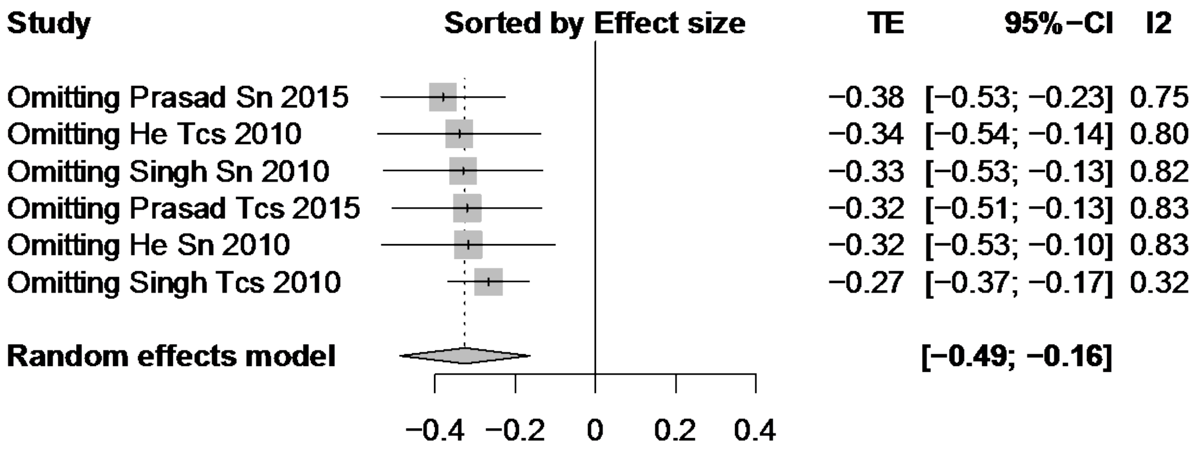

The plot is ordered by effect size (high to low). From the plot it can be derived that the outlying study Singh Tcs 2010 has very high effect sizes. The overall effect size is smallest when this study is removed.

S9B-1) Influence analysis of studies using the Digital Plaque Imaging Analysis (DPIA) for the experiments in which the toothbrush was used with and without an active dentifrice

```
Influential analysis (Random effects model)

                              MD             95%-CI  p-value
tau^2   I^2
Omitting Bellamy Sn 2011   -2.8933 [-4.5541; -1.2324]   0.0006
0.0000  0.0%
Omitting Bellamy Sn 2014   -2.9600 [-4.6106; -1.3093]   0.0004
0.0000  0.0%
Omitting Bellamy Sn 2009   -3.0827 [-4.6257; -1.5397] < 0.0001
0.0000  0.0%
Omitting White Sn 2007     -3.1126 [-4.7026; -1.5226]   0.0001
0.0000  0.0%
Omitting White Sn 2006     -3.1455 [-4.6909; -1.6001] < 0.0001
0.0000  0.0%
Omitting White Sn 2008     -3.2038 [-4.7217; -1.6859] < 0.0001
0.0000  0.0%
Omitting White Tcs 2008    -3.6022 [-5.1523; -2.0521] < 0.0001
0.0000  0.0%

Pooled estimate            -3.1513 [-4.6115; -1.6911] < 0.0001
0.0000  0.0%

Details on meta-analytical method:
- Inverse variance method
- DerSimonian-Laird estimator for tau^2
```

Calculating {Schwarzer, 2015 #6005} pooled estimates omitting one study at a time showed that no single study significantly influenced the pooled DiffMs.

S9B-2) Baujat Plot {Baujat, 2002 #6195} of overnight morning END plaque scores using the Digital Plaque Imaging Analysis (DPIA) for the experiments in which the toothbrush was used with and without an active dentifrice

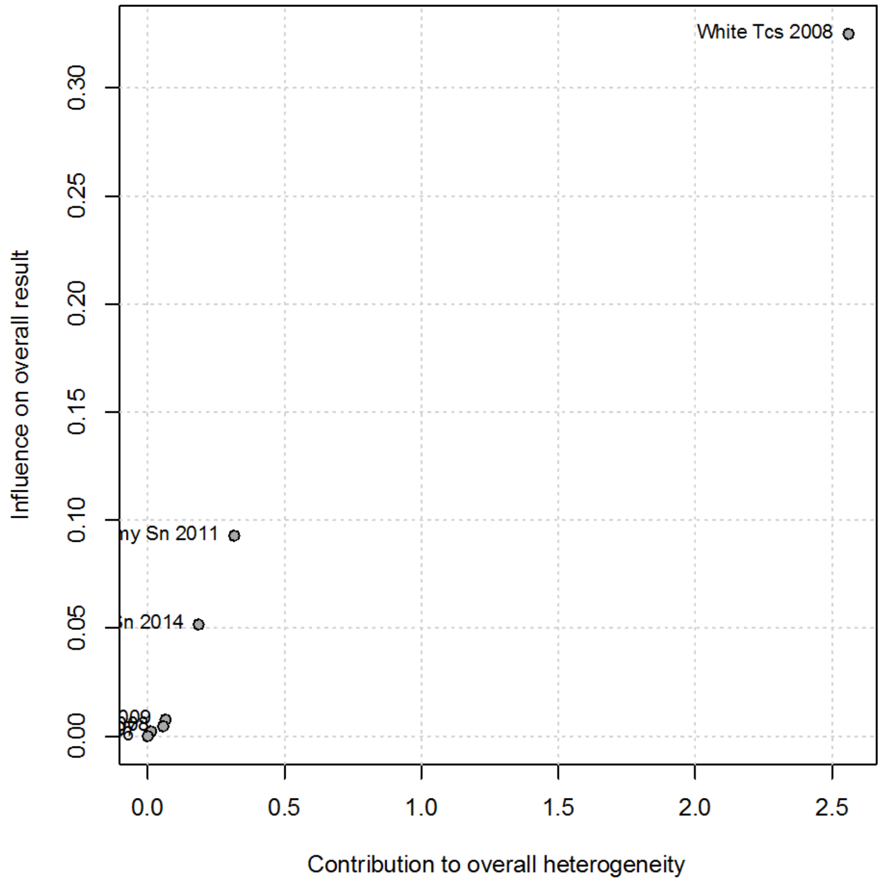

The plot shows that the experiment White Tcs 2008 contribute much to the overall heterogeneity and is also high influential concerning the overall pooled effect.

S9B-3) Galbraith Plot of overnight morning END plaque scores using the Digital Plaque Imaging Analysis (DPIA) {Sagel, 2000 #5682}for the experiments in which the toothbrush was used with and without an active dentifrice

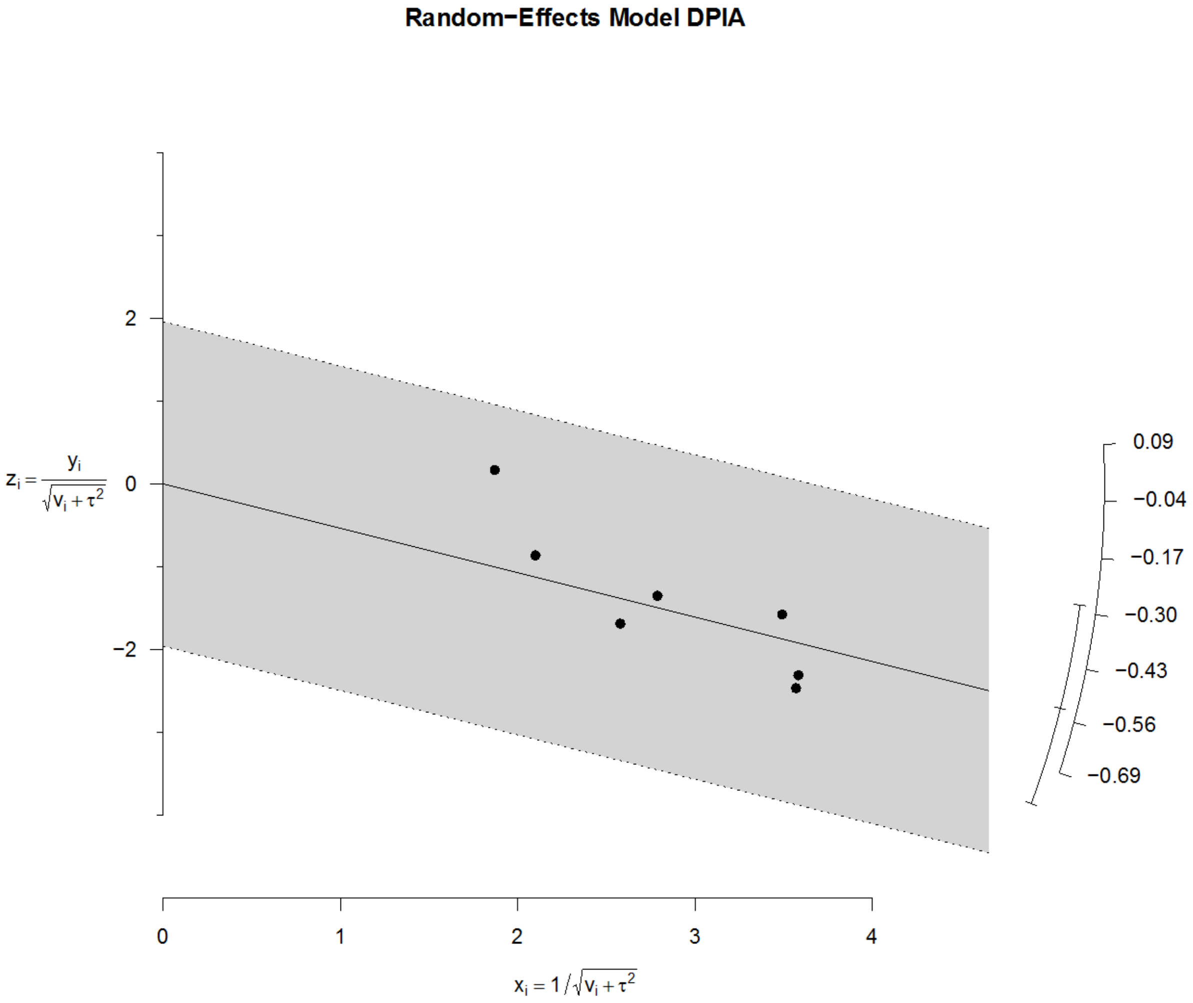

Galbraith's radial plot applied to data from END plaque scores. Each trial is plotted as a dot. Heterogeneous trials are visualized beyond the lines representing the approximated 95 per cent confidence limits.
The plot does not show specific trials as "problematic" sources of heterogeneity.

S9B-4a) Leave-One-Out-Analyses ordered by heterogeneity for the experiments using the Digital Plaque Imaging Analysis (DPIA){Sagel, 2000 #5682}

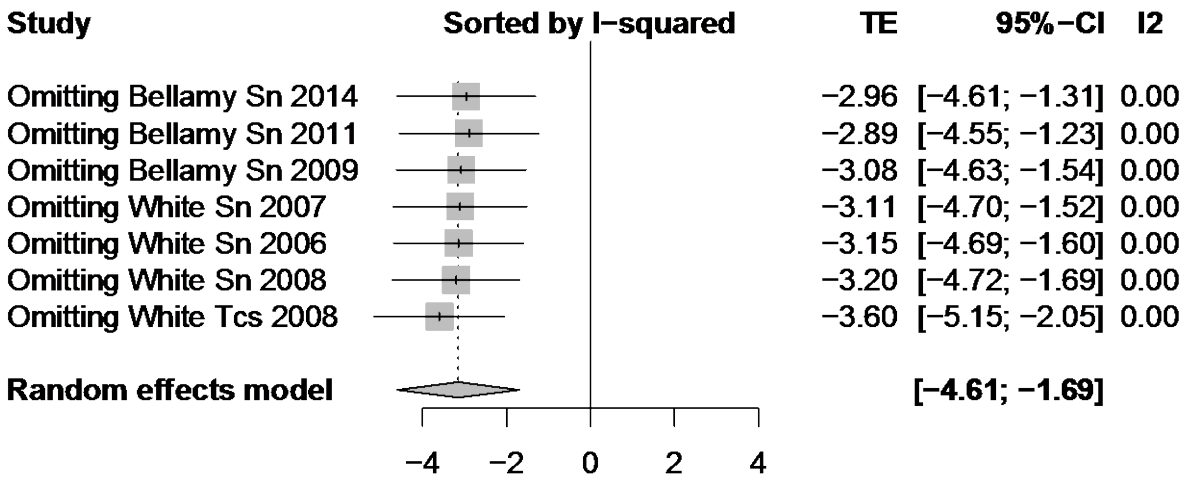

The plot is ordered by heterogeneity (low to high), as measured by I^2. From the plot it can be deduced that there is no effect on I^2 heterogeneity by omitting an experiment.

S9B-4b) Leave-One-Out-Analyses ordered by Effect size for the experiments using the Digital Plaque Imaging Analysis (DPIA){Sagel, 2000 #5682}

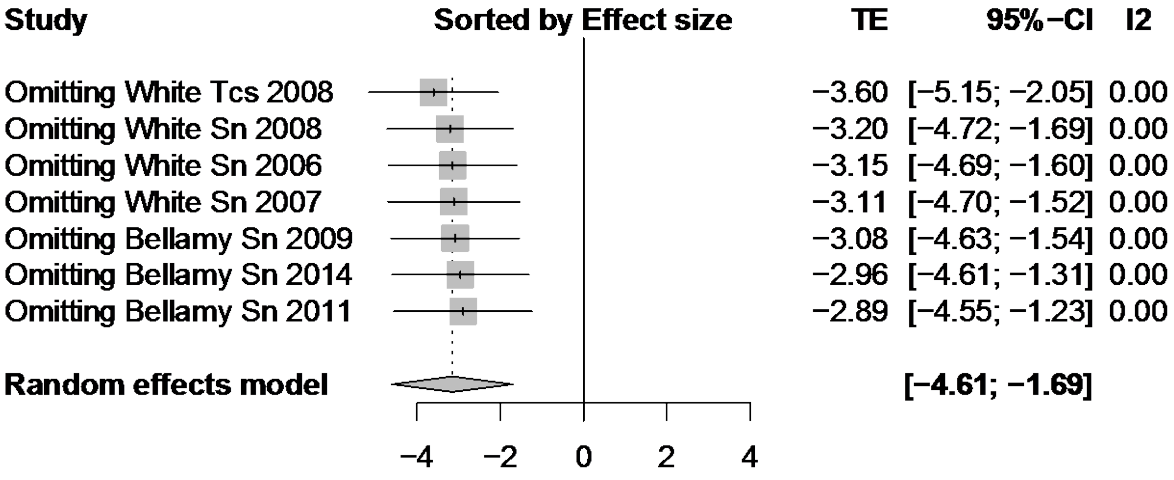

The plot is ordered by effect size (high to low). From the plot it can be derived that the outlying study White Tcs 2008 has high effect sizes but that there is no effect on I^2 heterogeneity by omitting this experiment.

Appendix S10 Trial Sequential Analysis

S10-1) TSA of the overnight morning END plaque scores using the Turesky et al. {Turesky, 1970 #5462}modification of the Q&H (1962) Plaque Index clinically for the experiments in which the toothbrush was used with and without an active dentifrice.
TSA suggests that the statistical evidence is firm for this meta-analysis. The number of participants does reach the information size and the cumulative Z-curve does cross the monitoring boundary.

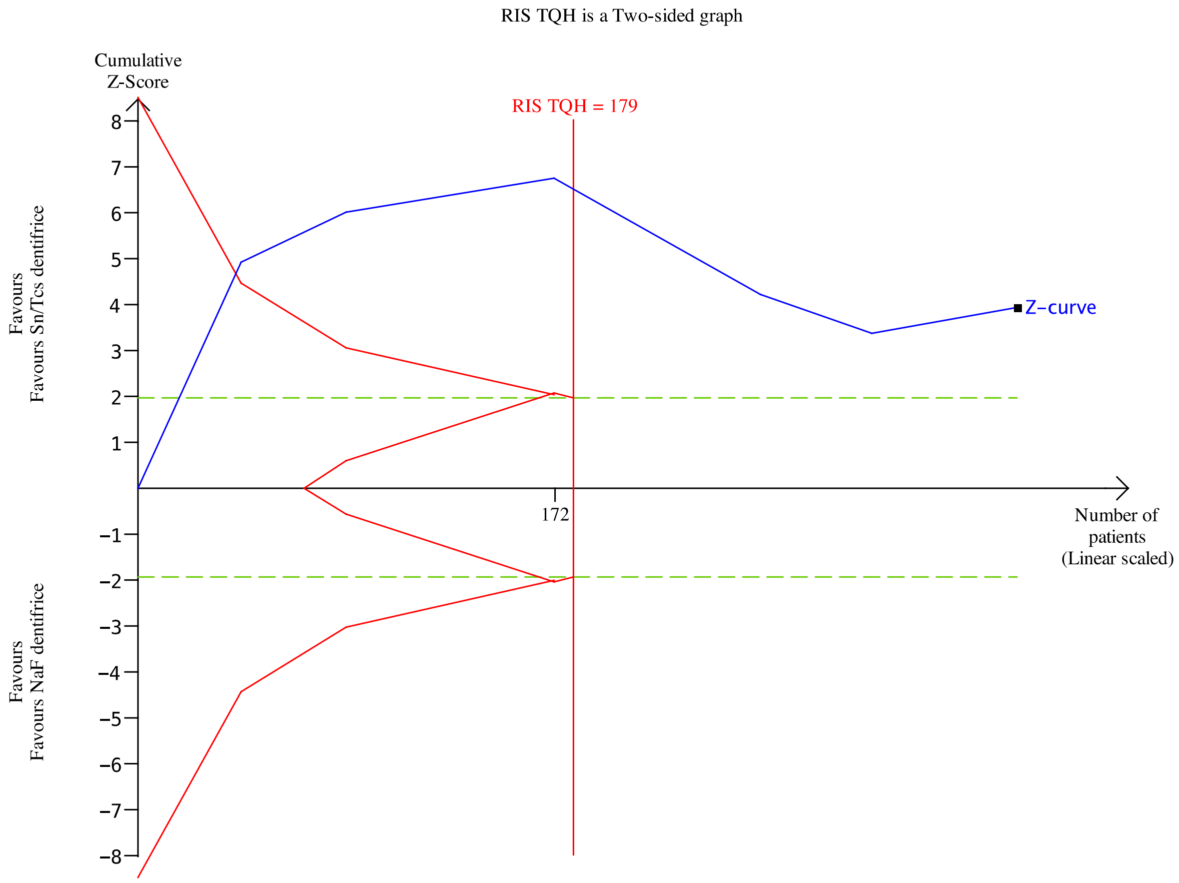

The cumulative blue Z-curves were constructed with each cumulative Z-value calculated after including a new trial according to publication date. Crossing of the two-sided Z = 1.96 provides a traditionally significant result. Crossing of the red trial sequential monitoring boundaries is needed to obtain reliable evidence adjusted for random error risk. Z-curves not crossing Z= 1.96 indicate absence of evidence if the information size is not reached or lack of the predefined intervention effect if the information size is not reached {Brok, 2008 #6066;Brok, 2008 #6066}. The green dotted lines represent the traditional boundary. The vertical red line represents the estimated heterogeneity-adjusted required information size, the number of participants for the meta-analysis sample size.

S10-2). TSA of the overnight morning END plaque scores using the DPIA Plaque Index {Sagel, 2000 #5682} clinically for the experiments in which the toothbrush was used without an active dentifrice and stannous fluoride as active dentifrice. TSA suggests that the statistical evidence is firm for this meta-analysis. The number of participants does reach the information size and the cumulative Z-curve does cross the monitoring boundary.

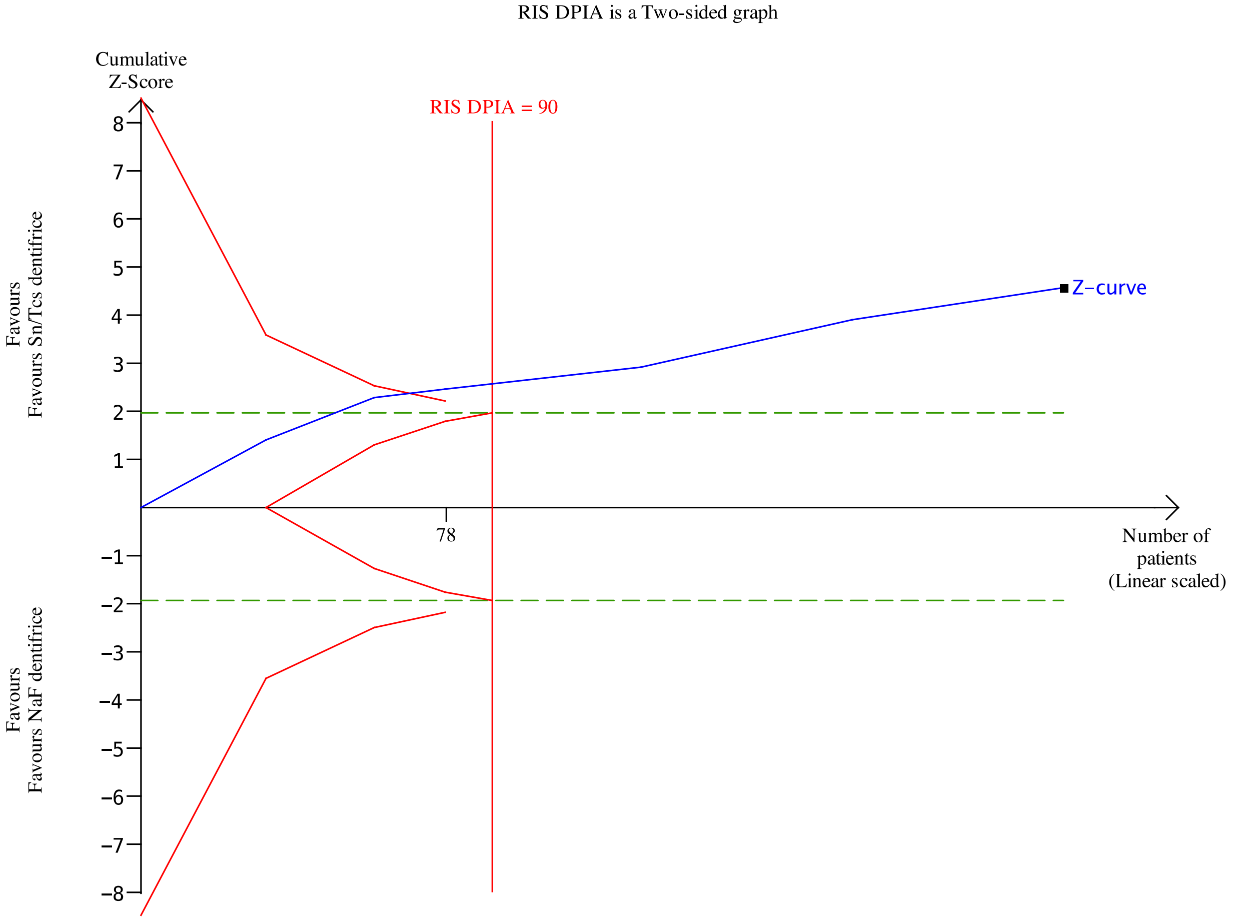

The cumulative blue Z-curves were constructed with each cumulative Z-value calculated after including a new trial according to publication date. Crossing of the two-sided Z = 1.96 provides a traditionally significant result. Crossing of the red trial sequential monitoring boundaries is needed to obtain reliable evidence adjusted for random error risk. Z-curves not crossing Z= 1.96 indicate absence of evidence if the information size is not reached or lack of the predefined intervention effect if the information size is not reached {Brok, 2008 #6066;Brok, 2008 #6066}. The green dotted lines represent the traditional boundary. The vertical red line represents the estimated heterogeneity-adjusted required information size, the number of participants for the meta-analysis sample size.

Remark: we considered the only experiment with triclosan {White, 2008 #6194} to be an outlier and excluded this experiment in the TSA. See for this reason also Appendix S8f-1.

Appendix S11

Post-hoc sensitivity analysis of the cross-over trials using the DPIA Plaque Index {Sagel, 2000 #5682} included in this MA. Sensitivity analysis with correlation values of 0, 0.25 and 0.5. The sensitivity analysis of the crossover trials with correlation coefficients of 0, 0.25 and 0.5 are in agreement with the results of the MA

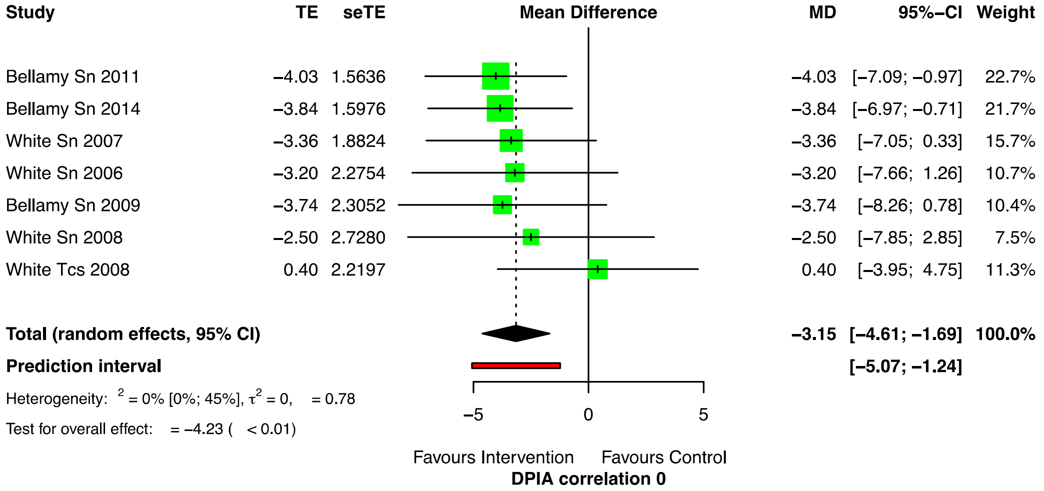

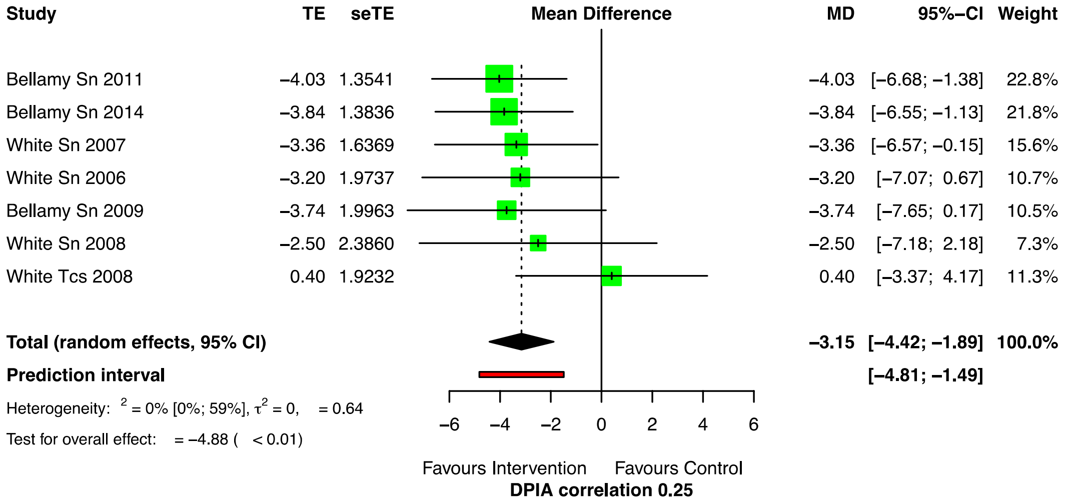

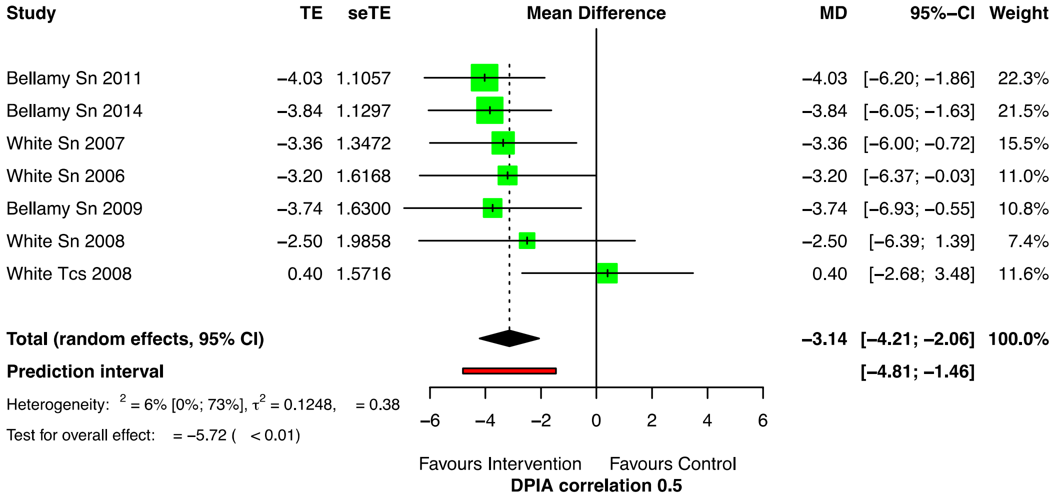

Appendix S12 Clinical relevance assessment of the overnight morning END plaque scores using the Turesky et al. {Turesky, 1970 #5462} modification of the Q&H (1962) Plaque Index clinically for the experiments in which the toothbrush was used with and without an active dentifrice

DATA ENTRY																	STANDARDIZED EFFECT SIZE					
Study	Outcome measure	Treatment group baseline			Control group baseline			Treatment group end			Control group end			Mean Difference	Confidence Interval for Difference		Effect Size (Cohen's d)	Bias corrected (Hedges)	Interpretation ES	MID (0.2)	MID (0.5)	Final decision clinical relevance
		mean	n	SD	mean	n	SD	mean	n	SD	mean	n	SD		lower	upper						
He 2010	Tcs-NaF	3.11	29	0.27	3.04	14	0.26	2.74	29	0.22	2.99	14	0.21	-0.25	-0.39	-0.11	-1.17	-1.15	LEF	0.05	0.13	CR
Singh 2010	Tcs-NaF	2.34	57	0.38	2.22	28	0.43	1.38	57	0.38	2.06	28	0.39	-0.68	-0.86	-0.50	-1.77	-1.76	LEF	0.08	0.20	CR
Prasad 2015	Tcs-NaF	2.39	42	0.54	2.30	18	0.53	1.48	42	0.48	1.83	18	0.52	-0.35	-0.63	-0.07	-0.71	-0.70	MES	0.11	0.27	CR
He 2010	Sn-NaF	3.11	29	0.27	3.04	14	0.26	2.65	29	0.22	2.99	14	0.212	-0.34	-0.48	-0.20	-1.59	-1.56	LEF	0.05	0.13	CR
Singh 2010	Sn-NaF	2.27	58	0.41	2.22	28	0.43	1.77	58	0.46	2.06	28	0.39	-0.29	-0.49	-0.09	-0.66	-0.65	MES	0.08	0.21	CR
Prasad 2015	Sn-NaF	2.26	28	0.63	2.30	18	0.53	1.89	28	0.52	1.83	18	0.52	0.06	-0.26	0.38	0.12	0.11	SES	0.12	0.30	NCR
**Pooled Tcs**	Tcs-NaF	2.53	128	0.42	2.435	60	0.431	1.72	128	0.39	2.208	60	0.40	-0.49	-0.61	-0.37	-1.24	-1.24	LEF	0.08	0.21	CR
**Pooled Sn**	Sn-NaF	2.4794	115	0.448	2.435	60	0.435	2.02	115	0.43	2.208	60	0.40	-0.19	-0.32	-0.05	-0.44	-0.44	MES	0.09	0.22	PCR

CR: clinically relevant; when both the calculated effect size (ES)* is ≥ 0.40 and the mean difference between groups are higher than both MIDs (minimal important differences) **

PCR: potentially clinically relevant; if ES is small/ moderate and one of the MIDs is accomplished

NCR: not clinically relevant; if ES is small and one of the MID is accomplished or if both (ES and MID) are not accomplished or clinical criterion determines NCR

MID: minimal important difference; a mean difference between groups that is higher than the MID can be considered as clinically relevant (Lemieux 2007, Musselman 2007).

SES: small effect size; 0.20 (0-0.39)

MES: medium effect size; 0.50 (0.4-0.79)

LEF: large effect size; ≥0.80

***** Effect sizes (ES) according to Cohen {Cohen, 1988 #6232}

** MID(0.2)= 0.2 x pooled SD; MID(0.5)= 0.5 x pooled SD

Appendix S13

Limitations related to the evidence that emerges from this review.

Several limitations were identified for this review.

- While there is an emerging evidence base in public health, the evidence can often be difficult to find. Indexing of journals in MEDLINE has assisted those conducting systematic reviews to more easily identify published studies. However, information technology and the processes associated with indexing are not infallible. Studies may not be correctly marked by study design which may mean they are missed in the electronic searching process [h].

- The more resources searched, the higher the yield, and thus time and costs required to conduct a systematic review. While there is an abundance of evidence to suggest how extensive a search for randomized controlled trials (RCTs) should be, it is neither conclusive nor consistent [i].

- Another limitation may be the use of published research papers only. No effort was made to retrieve information from industry on unpublished data. The authors of this review did not have the resources to obtain data that are kept 'on file' by the various dentifrice manufacturers. This is known as the 'file drawer problem', as a form of publication bias [j,k].

- Due to the focused question of this SR, no long-term studies were involved. Longer-duration studies of antimicrobial properties of dentifrice will be more representative of home-use circumstances [l].

- The compliance of the given protocols may be considered as an important factor in the study outcomes. None of the studies mentioned that compliance was evaluated.

- Various toothbrush types were used in the studies included and therefore evaluation of the added benefit of the dentifrice between studies might be influenced by this diversity.

- The populations selected for studies of dental plaque assessment, in most cases, would be xindividuals with mild to moderate gingivitis [m]. The question is whether it corresponds to the average person in the population. It is quite conceivable that some people with significant plaque formation benefit substantially more from a dentifrice with active ingredients than individuals do with little plaque formation.

- The clinically subjective indices are limited for two primary reasons: inconsistent application of the index, especially in long clinical trials, often leads to greater variation in the data and sensitivity of the scale often leads to larger studies required to define averages [n].

- All the included studies became available during the last two decades. However, in the majority of cases, the manner of reporting did not follow current standards, such as CONSORT 2010 and TIDieR 2014. This limitation is also reflected in the results of the risk of bias assessment. This systematic review reinforces the importance of correct and complete reporting and adherence to standards, particularly the new TIDieR checklist regarding the description and replication of interventions [o].

Appendix S13 Post hoc changes to the protocol

- The Cochrane Collaborations Methodological Expectations of Cochrane Intervention Reviews (MECIR) guidelines state that searching MEDLINE, EMBASE (if available to the review author) and CENTRAL should be considered mandatory [p]. Therefore, EMBASE has also been used.
- For reasons that we want to search for articles as completely as possible, we have used Google Scholar as an additional source. As a result, Google Scholar allowed to identify two additional studies.

Appendix S14 Abbreviations

Active Dentifrice	Stannous or Triclosan Dentifrice
ADF	Active Dentifrice
AmF	Amine fluoride
CHX	Chlorhexidine
C.V.	C. Valkenburg
D.E.S.	D.E.Slot
DPIA	Digital Plaque Imaging Analysis
G.A.W.	G.A. van der Weijden
MFP	Sodium Monofluorophosphate
NaF	Sodium fluoride
NaF/KNO3	Sodium fluoride/ potassium nitrate
O/RB	Oscillating-rotating power toothbrush
PVM/MA	Polyvinylmethyl ether/maleic acid
RDA	Relative (or Radioactive) Dentine Abrasivity (or Abrasion)
RDF	Regular (sodium fluoride) Dentifrice
Regular Dentifrice	Sodium fluoride dentifrice
SLS	Sodium Lauryl Sulfate
SMFP	Sodium monofluorophosphate
Sn	Stannous Dentifrice
SnDF	Stannous Dentifrice
SnF2	Stannous fluoride
Tcs	Triclosan Dentifrice
TDF	Test Dentifrice
TQH	Turesky Modification of the Quigley-Hein Plaque Index

Additional references:

a. Zhang Z, Kossmeier M, Tran US, Voracek M, Zhang H. Rainforest plots for the presentation of patient-subgroup analysis in clinical trials. Ann Transl Med. 2017;5:485.

b. https://github.com/Mkossmeier/metaviz

c. Zhang Z, Kossmeier M, Tran US, Voracek M, Zhang H. Rainforest plots for the presentation of patient-subgroup analysis in clinical trials. Ann Transl Med. 2017;5:485.

d. https://cran.r-project.org/web/packages/metaviz/metaviz.pdf

e. Beath KJJRJ. metaplus: An R Package for the Analysis of Robust Meta-Analysis and Meta-Regression. 2016;8.

f. Lin L, Chu H, Hodges JS. Alternative measures of between-study heterogeneity in meta-analysis: Reducing the impact of outlying studies. Biometrics. 2017;73:156-166.

g. Barnett V, Lewis T. Outliers in statistical data. 3rd ed. New York, NY: John Wiley & Sons; 1974.

~~h. Armstrong R, Jackson N, Doyle J, Waters E, Howes F. It's in your hands: the value of handsearching in conducting systematic reviews of public health interventions. Journal of public health. 2005;27:388-391.~~

~~i. Crumley ET, Wiebe N, Cramer K, Klassen TP, Hartling L. Which resources should be used to identify RCT/CCTs for systematic reviews: a systematic review. BMC Med Res Methodol. 2005;5:24.~~

~~j. Rosenthal R. The file drawer problem and tolerance for null results. Psychol Bull. 1979;86:638.~~

~~k. Keukenmeester RS, Slot DE, Putt MS, Van der Weijden GA. The effect of medicated, sugar-free chewing gum on plaque and clinical parameters of gingival inflammation: a systematic review. Int J Dent Hyg. 2014;12:2-16.~~

~~l. Slot DE, Wiggelinkhuizen L, Rosema NA, Van der Weijden GA. The efficacy of manual toothbrushes following a brushing exercise: a systematic review. Int J Dent Hyg. 2012;10:187-197.~~

~~m. American Dental Association (ADA). Council on Scientific Affairs. Acceptance Program Requirements: Adjunctive Dental Therapies for the Reduction of Plaque and Gingivitis. 2011;19.~~

~~n. Sagel PA, Lapujade PG, Miller JM, Sunberg RJ, P.A S, P.G L, et al. Objective quantification of plaque using digital image analysis. Monogr Oral Sci. 2000;17:130--143.~~

~~o. Hoffmann TC, Glasziou PP, Boutron I, Milne R, Perera R, Moher D, et al. Better reporting of interventions: template for intervention description and replication (TIDieR) checklist and guide. BMJ. 2014;348:g1687.~~

h. Chandler J, Churchill R, Higgins J, Lasserson T, Tovey D. Methodological standards for the conduct of new Cochrane Intervention Reviews. The Cochrane Library. 2013.
